# Gastric cancer cell-originated small extracellular vesicle induces metabolic reprogramming of BM-MSCs through ERK-PPARγ-CPT1A signaling to potentiate lymphatic metastasis

**DOI:** 10.1186/s12935-023-02935-5

**Published:** 2023-05-09

**Authors:** Jiaying Huang, Xiang Wang, Jing Wen, Xinxin Zhao, Chen Wu, Lin Wang, Xiaoli Cao, Haibo Dong, Xuejing Xu, Feng Huang, Wei Zhu, Mei Wang

**Affiliations:** 1 Department of Laboratory Medicine, School of Medicine, Jiangsu University, 301 Xuefu Road, Zhenjiang, 212013 Jiangsu Province China; 2 Department of Laboratory Medicine, Affiliated Tumor Hospital of Nantong University, Nantong, Jiangsu Province China; 3 Department of Hematology, Nanjing Drum Tower Hospital, Affiliated Hospital of Jiangsu University, 321 Zhongshan Road, Gulou District, Nanjing, Jiangsu Province China; 4 Department of Laboratory Medicine, Nanjing Drum Tower Hospital, Affiliated Hospital of Jiangsu University, 321 Zhongshan Road, Gulou District, Nanjing, Jiangsu Province China; 5 Department of Clinical Laboratory, Affiliated Kunshan Hospital of Jiangsu University, Suzhou, Jiangsu Province China; 6 Department of Clinical Laboratory, Maternal and Child, Health Care Hospital of Kunshan, Suzhou, Jiangsu Province China

**Keywords:** Mesenchymal stem cells, Gastric cancer, Lymph node metastasis, Small extracellular vesicles

## Abstract

Tumor microenvironment and metabolic reprogramming are critical for tumor metastasis. Bone marrow-derived mesenchymal stem cells (BM-MSCs) are widely involved in the formation of tumor microenvironment and present oncogenic phenotypes to facilitate lymph node metastasis (LNM) in response to small extracellular vesicles (sEV) released by gastric cancer (GC) cells. However, whether metabolic reprograming mediates transformation of BM-MSCs remains elusive. Herein, we revealed that the capacity of LNM-GC-sEV educating BM-MSCs was positively correlated with the LNM capacity of GC cells themselves. Fatty acid oxidation (FAO) metabolic reprogramming was indispensable for this process. Mechanistically, CD44 was identified as a critical cargo for LNM-GC-sEV enhancing FAO via ERK/PPARγ/CPT1A signaling. ATP was shown to activate STAT3 and NF-κB signaling to induce IL-8 and STC1 secretion by BM-MSCs, thereby in turn facilitating GC cells metastasis and increasing CD44 levels in GC cells and sEV to form a persistent positive feedback loop between GC cells and BM-MSCs. The critical molecules were abnormally expressed in GC tissues, sera and stroma, and correlated with the prognosis and LNM of GC patients. Together, our findings uncover the role of metabolic reprogramming mediated BM-MSCs education by LNM-GC-sEV, which presents a novel insight into the mechanism underlying LNM and provides candidate targets for GC detection and therapy.

**Supplementary Information:**

The online version contains supplementary material available at 10.1186/s12935-023-02935-5.

## Introduction

Gastric cancer (GC) ranks fifth in incidence and fourth in mortality worldwide [1], and is frequently diagnosed with regional lymph node metastasis (LNM). For patients at early-stage GC, LNM is associated with poor prognosis and even predicts distant metastasis [2, 3]. Thus, it is important to illustrate the molecular mechanisms underlying LNM in order to provide promising targets for clinical therapy. Bone marrow-derived mesenchymal stem cells (BM-MSCs) have been demonstrated to be recruited to tumor sites and contribute to the formation of tumor microenvironment (TME). MSCs as a vital type of stromal cells are widely reported to influence the malignant behaviors of GC cells [4, 5].

It is well established that tumor cells-derived small extracellular vesicles (sEV) are able to educate BM-MSCs to assist tumor growth and promote metastasis [6, 7]. For instance, sEV derived from lung cancer cells induce a pro-inflammatory phenotype in MSCs [6]. Cholangiocarcinoma sEV educate MSCs to modulate the microenvironment and promote tumor growth [7]. Previously, we have demonstrated that LNM-GC cell-sEV (LNM-GC-sEV) could induce the transition of BM-MSCs into tumor-associated MSCs, thus promoting GC progression [8]. Metabolism is a major regulator of cell fate and functions. Recently, emerging roles of fatty acid oxidation (FAO) metabolic reprogramming of stromal cells in tumor metastasis have caused concern. In a glucose-limited tumor microenvironment, enhanced FAO of cancer-associated fibroblasts (CAFs) promotes peritoneal metastasis of colon cancer [9]. FAO activation in CD8^+^ T effector cells is crucial for obesity-promoted breast tumor development [10]. CD44 was identified to be highly loaded in LNM-GC-sEV by Label-free proteomics and mediated malignant phenotype transmission from LNM-GC cells to primary GC cells by regulating FAO [11]. Furthermore, compared to BM-MSCs, primary GC tissues-derived MSCs (GC-MSCs) displayed enhanced FAO, which was pivotal for GC-MSCs facilitating GC metastasis (data unpublished). Therefore, whether LNM-GC-sEV educates BM-MSCs through FAO metabolic reprogramming is worthy of investigation.

Herein, we observed that the capacity of LNM-GC-sEV educating BM-MSCs was positively correlated with the LNM capacity of GC cells themselves. Enhanced FAO was required for the BM-MSCs education by LNM-GC-sEV. Mechanistically, CD44 was identified as a critical cargo for LNM-GC-sEV enhancing FAO by regulating ERK/PPARγ/CPT1A signaling. ATP might be the effective metabolite of FAO, which activated STAT3 and NF-κB signaling to induce the release of IL-8 and STC1 by BM-MSCs, thereby in turn promoting GC cells metastasis and increasing CD44 levels in GC cells and sEV. The key molecules involved in FAO metabolic reprogramming of BM-MSCs were abnormally expressed in GC and correlated with prognosis and LNM of GC patients, and might be explored as diagnostic and prognostic indicators and therapeutic targets for GC.

## Methods

### Cell culture and treatment

Human GC cell lines (AGS and HGC-27) and lymphatic capillary endothelial cells (HLECs) were purchased from Procell Life Science&Technology Co., Ltd (Wuhan, China). GC cells with highly lymphatic metastases (HGC-27L) were obtained by continuous passage in vivo as previous description [11]. AGS was cultured in DMEM / Ham’s F-12 (1:1) (DF-12) (Gibco, Thermo Fisher Scientific, Inc., MA, USA) containing 10% fetal bovine serum (FBS) (SERANA, Brandenburg, Germany). HGC-27 and HGC-27L were cultured in RPMI-1640 (Gibco) with 10% FBS. HLECs were maintained with Dulbecco’s Modified Eagle Medium (Gibco) containing 10% FBS. Cells were cultured at 37 °C with 5% CO_2_. BM-MSCs and GC-MSCs were obtained and cultured as previously described [12]. BM-MSCs were plated in a six-well plate at a cell density of 4 × 10^4^ per well and incubated with 100 μg/mL sEV for 48 h after attachment overnight. Etomoxir (Cat. No. HY-50202, MedChemExpress, NJ, USA), U0126 (Cat. No. S1901, Beyotime Biotechnology, Shanghai, China) and GW9662 (Cat. No. HY-16578, MedChemExpress) were dissolved in DMSO at a concentration of 10 mM and stored at − 80 °C. BM-MSCs were pretreated with etomoxir (100 μM), U0126 (20 μM) and GW9662 (10 μM) for 24 h, and then incubated with sEV for 24 h. BM-MSCs were treated with Cycloheximide (CHX) (Cat. No. SC0353, Beyotime Biotechnology) at final concentration of 15 μg/mL alone or together with HGC-27L-sEV for 6 h. BM-MSCs were treated with 5 µmol/L ATP (Cat. No. HY-B2176, MedChemExpress) for 30 min after culture in α-Minimum Essential Medium (α-MEM) for 24 h. For IL-8 and STC1 neutralization experiments, IL-8 neutralizing antibody (1.5 μg/mL, MAB208, R&D Systems, USA), the conditioned media (CM) obtained from BM-MSCs were incubated with STC1 antibody (1 μg/mL, 20621-1-AP, Proteintech, China) and equivalent species IgG at room temperature for one hour and were then used to treat GC cells.

### Clinical specimens

Clinical tissues were obtained from the Affiliated Hospital of Jiangsu University. Sera of normal subjects and GC patients were collected from the Affiliated Tumor Hospital of Nantong University. All procedures were approved by the Ethics Committee of the above hospitals and Jiangsu University. Patient informed consent was obtained before sample collection.

### Isolation and characterization of sEV

GC cells were cultured in medium containing 10% sEV-depleted FBS for 48 h. The supernatant of GC cells was harvested and centrifuged at 2000 g for 20 min at 4 °C to remove the cell fragments. The cell-free supernatant was ultra-centrifuged at 100,000 g for 70 min to collect sEV pellet. The isolated sEV were washed with phosphate buffered saline (PBS) and stored at − 80 °C. The protein concentration of sEV was measured by BCA assay (Beyotime Biotechnology). Transmission electron microscopy (TEM), nanoparticle tracking analysis (NTA) and western blotting were performed to characterize sEV as described previously [13].

### Cell transfection

Small interfering RNAs (siRNAs) against CD44 (si-CD44) and their corresponding negative control oligonucleotide (NC) were purchased from GenePharma (Shanghai, China). HGC-27L cells were transfected with the indicated oligonucleotide by Lipofectamine 2000 (Invitrogen, Thermo Fisher Scientific, Inc., MA, USA). The sequences of oligonucleotides were present in Additional file 1: Table S1. Lentivirus was purchased from Genechem (Shanghai, China). AGS cells were infected with the CD44-overexpressing lentivirus (pLV-CD44) and vector virus (pLV-vector) at the multiplicity of infection (MOI) of 15.

### Immunofluorescence assay

Immunofluorescence staining was conducted with anti-α-SMA (BM0002, Boster Biological Technology, China) as described previously [13]. α-SMA expression in BM-MSCs was observed under a fluorescence microscope (Zeiss AG, Oberkochen, Germany).

### Transwell assay

Transwell assay was performed as previous description [8]. Briefly, the lower chamber was supplemented with medium containing 10% FBS. For migration assay, GC cells (8 × 10^4^ per well) suspended in serum-free medium were added to the upper chamber followed by incubation at 37 °C for 10 h. For invasion assay, the same number of GC cells were added to the upper chamber precoated with matrigel (Cat. No. 354234, BD Bioscience, San Jose, CA, USA) and incubated at 37 °C for 24 h. The cells on the lower surface of the upper chamber were stained with crystal violet and captured using the microscope.

### Tubule formation analysis

HLECs (3 × 10^4^ per well) were added into the 96-well plate pre-coated with 50 μL matrigel (BD Bioscience) and incubated at 37 °C for 5 h. Tubule formation was observed using a microscope and the junction number was counted by Image J software.

### Western blotting

The protocol and procedure for western blotting were as described previously [14]. Primary antibody anti-CPT1A (#12252) was obtained from Cell Signaling Technology (Danvers, USA). Primary antibodies anti-GAPDH (MB9231), anti-CD9 (BS60359) and anti-CD63 (BS72936) were purchased from Bioworld Technology (Minnesota, USA). Primary antibodies anti-CD44 (A12410), anti-STAT3 (A1192), anti-Phospho-STAT3-Y705 (AP0070), anti-NF-kB p65/RelA-S536 (A2547), anti-Phospho-NF-kB p65 (AP0475), anti-Calnexin (A15631), the secondary antibodies HRP goat anti-rabbit IgG (AS014) and goat anti-mouse IgG (AS003) were purchased from ABclonal Technology (Wuhan, China).

### Quantitative real-time PCR (*q*PCR) analysis

Extraction of total RNA was performed using TRIzol reagent (Invitrogen). The reverse transcription was conducted by PrimeScript™ RT reagent KIT (Takara, Dalian, China). *q*PCR was performed using TB Green^®^ Premix Ex TaqTM II kit (Takara) following the manufacturer’s procedure. The sequences of primers are listed in Additional file 2: Table S2.

### Transcriptome sequencing

The total RNA of the BM-MSCs treated with NC-sEV and si-CD44-sEV was extracted and delivered to the Genedenovo Biotechnology Company (Guangzhou, China) for transcriptome sequencing. EdgeR and DESeq2 software were used to identify the differential expression genes with *p*-value < 0.05 and fold changes ≥ 2. Gene Set Enrichment Analysis (GSEA) was conducted to characterize the potential mechanisms regarding the impact of CD44 on paracrine regulation by BM-MSCs.

### ELISA

ELISA was conducted using Human CD44/Heparan Sulfate Proteoglycan ELISA KIT (RayBiotech Life, Inc., GA, USA), Human IL-8 ELISA KIT (RayBiotech Life, Inc., GA, USA) and Human STC1 ELISA KIT (RayBiotech Life, Inc., GA, USA) following the manufacturer’s procedure.

### Immunohistochemistry (IHC) assay

IHC was performed with primary antibodies against CD105 (A19008, ABclonal, China), CD44 (A12410, ABclonal, China), CPT1A (#12252, CST, USA), pan-cytokeratin (AE1/AE3) (ab27988, Abcam, UK), IL-8 (A2541, ABclonal, China) and STC1 (20621-1-AP, Proteintech, China) using instant SABC-POD Kit (Boster Biological Technology) following the manufacturer’s procedure. The samples were scanned and imaged using the Automatic digital slice scanning system. The staining scores of target proteins were obtained by the percentage of positive cells (< 5%: 0, 5–25%: 1, 26–50%: 2, 51–75%: 3, 76–100%: 4) multiplied by the staining intensity of positive cells (weak: 1, moderate: 2, strong: 3).

### β-oxidation rate assay

The cell mitochondria of BM-MSCs with different treatments were isolated integrally with the Cell Mitochondrial Isolation Kit (Beyotime, Shanghai, China). Then the mitochondria were used for β-oxidation rate assay according to the instruction of the Fatty Acid β-Oxidation Kit (GenMed Scientifics Inc., USA).

### Measurement of ATP and the activity of CPT1

BM-MSCs with indicted treatment were digested using trypsin and collected for ATP level and CPT1 activity assay by using Enhanced ATP Assay Kit (Beyotime, Shanghai, China) and Carnitine palmityl transferase1 (CPT1) kit (Ziker, Shenzhen, China), respectively.

### Animal model

Five to seven-week-old BALB/c male nude mice were purchased from the Changzhou Cavens Laboratory Animal Company (Changzhou, China) for the establishment of LNM model. Cell suspension containing 2 × 10^6^ of HGC-27 treated with indicated BM-MSC-CM was injected into the footpad of nude mice. Three or four weeks later, mice were sacrificed and the popliteal LNs were harvested to weigh and perform IHC assay. All the experiments on animals were performed with the approval of the Committee on the Use and Care of Animals of Jiangsu University.

### Bioinformatics analysis

The UCSC Xena (https://xenabrowser.net/datapages/) was used to download the RNA sequencing data of GC tissue in TCGA database and normal tissue in GTEx database. The data were analyzed by the software R (Version 3.6.3) with ggplot2 (Version 3.3.3). Cox proportional-hazards regression was performed by the software R (Version 3.6.3) with survival package (Version 3.2-10). Kaplan–Meier Plotter (https://kmplot.com/analysis/) was used to analyze the association of related genes with overall survival (OS) of GC patients.

### Statistical analysis

Statistical analyses were performed by GraphPad Prism 5 software. Data are presented as mean ± standard deviation (SD), unless separately stated. All experiments were performed in triplicate. Independent T test was used to analyze the differences between the two groups. One-way ANOVA was used for comparisons among multiple groups. Wilcoxon rank sum test was used to explore the differential expression of related genes between GC tissue and normal tissue. Spearman was used to analyze the correlations between related genes. Univariate and multivariate analyses were conducted by Cox regression. Relative risks were expressed as odds ratios with 95% confidence interval (CI). *P* < 0.05 indicates statistically significance.

## Results

### The ability of LNM-GC-sEV to educate BM-MSCs is determined by the lymphatic metastatic capacities of GC cells themselves

**Fig. 1:**
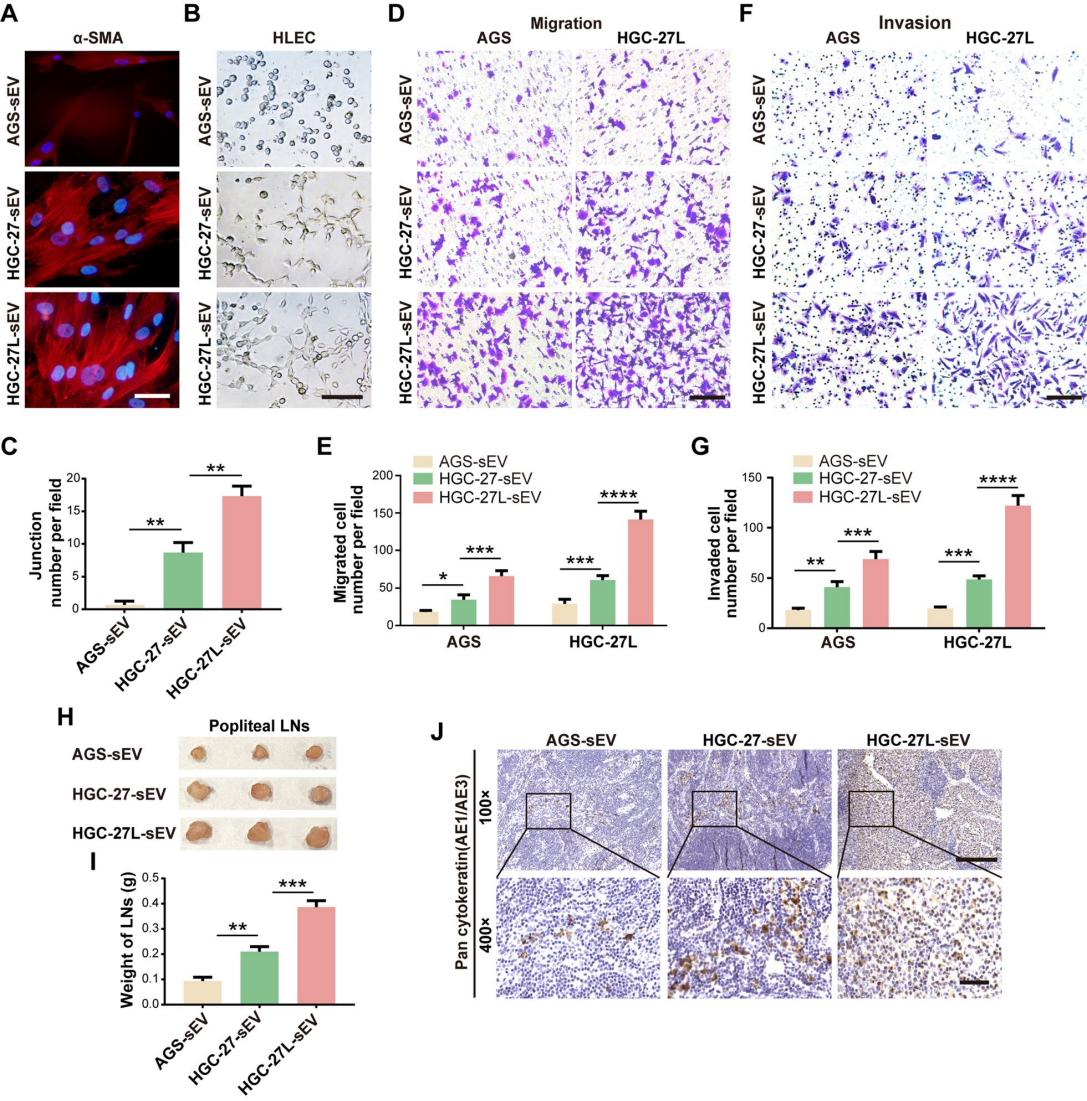
The ability of LNM-GC-sEV to educate BM-MSCs is determined by lymphatic metastatic capacities of GC cells themselves. **A-G** Comparison of the ability of AGS-sEV, HGC-27-sEV and HGC-27L-sEV to educate BM-MSCs in vitro. **A** α-SMA expression in BM-MSCs treated with different GC-sEV was measured by immunofluorescence staining (scale bars, 100 μm). **B, C** Morphology and quantitative analysis of tubule formation (scale bar, 200 μm). **D-G** Morphology and count of migrated and invaded cells (scale bar, 100 μm). **H-J** The tumor-promoting capacity of the above treated BM-MSCs was evaluated in vivo*.* **H** The harvested popliteal LNs. **I** Weight of popliteal LNs. **J** Representative images of pan-cytokeratin (AE1/AE3) staining in the popliteal LNs. (Top: scale bars, 100 μm; Bottom: scale bars, 20 μm). *P < 0.05; **P < 0.01; ***P < 0.001; ****P < 0.0001

sEV was individually isolated from AGS (primary GC cells), parental HGC-27 (LNM-GC cells) and an established HGC-27L with highly LNM capacity. All of GC-sEV displayed a typical saucer-like structure with a size range of 50-150 nm (Additional file 5: Fig. S1A, B) as well as positive expression of typical exosomal markers CD9 and CD63 (Additional file 5: Fig. S1C). BM-MSCs were then incubated with the above isolated GC-sEV. It has been widely reported that α-SMA is a typical marker of activated tumor-associated stromal cells [7, 15]. Immunofluorescence assay revealed that α-SMA expression intensity was gradually increased in BM-MSCs in HGC-27-sEV group and HGC-27L-sEV group compared to those in AGS-sEV group (Fig. 1a). Moreover, CM from the above treated BM-MSCs was harvested to treat HLEC and GC cells. The in vitro functional analysis showed that the abilities of the above treated BM-MSCs to promote HLEC tubule formation, GC cells migration and invasion were gradually enhanced by HGC-27-sEV and HGC-27L-sEV (Fig. 1b–g). In vivo, HGC-27 cells incubated with the above treated BM-MSCs-CM were used to establish LNM model. The volume, weight and pan-cytokeratin AE1/AE3 expression of the harvested popliteal lymph nodes were increased sequentially in AGS-sEV group, HGC-27-sEV group and HGC-27L-sEV group (Fig. 1h–j). These findings indicate that the ability of sEV to educate BM-MSC is positively correlated with the LNM capacity of the source GC cells.

### Enhanced FAO is required for BM-MSCs education by LNM-GC-sEV

**Fig. 2:**
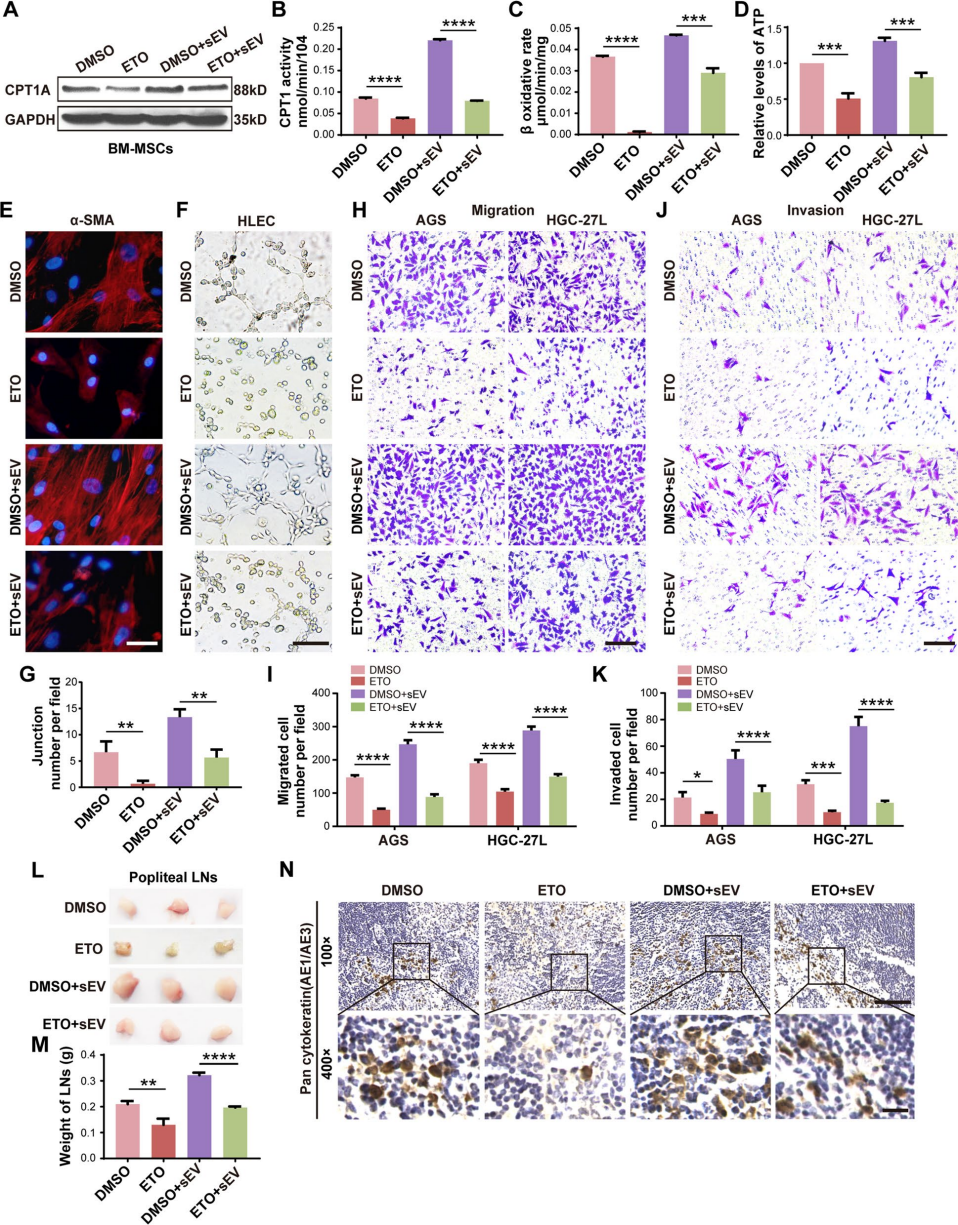
Enhanced FAO is required for BM-MSCs education by LNM-GC-sEV. **A** CPT1A was detected by western blotting in BM-MSCs treated with etomoxir and then incubated with GC-sEV. **B–D** The activity of CPT1, β-oxidation rate and ATP level of the above treated BM-MSCs were measured. **E–K** In vitro analysis of the effects of etomoxir on the education of BM-MSCs by GC-sEV. **E** Immunofluorescence staining for α-SMA. **F, G** Tubule formation assay. **H–K** Migration and invasion assay. **L–N** In vivo analysis of the effect of etomoxir on the tumor-promoting capacity of BM-MSCs induced by GC-sEV. **L** The harvested popliteal LNs. **M** Weight of the popliteal LNs. **N** IHC analysis of pan-cytokeratin (AE1/AE3) expression in popliteal LNs. **P < 0.01; ***P < 0.001; ****P < 0.0001

Recently, metabolism reprogramming emerges to mediate stromal cells remodeling to promote metastasis [16]. To explore whether FAO was involved in BM-MSCs education by LNM-GC-sEV, CPT1A expression, CPT1 activity, β-oxidation rate and ATP level were detected to determine FAO activity in BM-MSCs after treated with different GC-sEV. As shown, FAO activity was notably elevated in BM-MSCs treated with HGC-27-sEV and HGC-27L-sEV compared to those treated with AGS-sEV (Additional file 6: Fig. S2A–E). Among the three groups, FAO levels were the most in HGC-27L-sEV group. To determine whether FAO was indispensable for BM-MSCs education by LNM-GC-sEV, FAO inhibitor etomoxir was used to pretreat BM-MSCs before incubation with HGC-27L-sEV. The results showed that etomoxir significantly blocked HGC-27L-sEV-induced CPT1A expression, CPT1 activity, β-oxidation rate and ATP level in BM-MSCs (Fig. 2a–d, Additional file 6: Fig. S2F). Meanwhile, although the expression of α-SMA in BM-MSCs was strikingly up-regulated by HGC-27L-sEV, etomoxir significantly reversed this effect (Fig. 2e). Etomoxir also eliminated the promotion of HLEC tubule formation, GC migration and invasion mediated by HGC-27L-sEV treated BM-MSCs (Fig. 2f–k). In vivo, etomoxir treatment resulted in smaller and lighter popliteal lymph nodes and lower positive rate of pan-cytokeratin AE1/AE3 even though BM-MSCs were treated with HGC-27L-sEV (Fig. 2l–n). The above results demonstrate that LNM-GC-sEV educates BM-MSCs depending on FAO metabolic reprogramming.

### Knockdown of CD44 abrogates LNM-GC-sEV prompting FAO metabolic reprogramming of BM-MSCs

Previously, we demonstrated that CD44 was highly enriched in LNM-GC-sEV by proteomic analysis. sEV-CD44 content was validated to be correlated with the lymphatic metastatic capacity of GC cells and stimulate FAO in the receipt primary AGS cells to transmit malignant phenotype [11]. We hypothesized that LNM-GC-sEV was more likely to increase FAO activity in BM-MSCs through CD44. In accordance with the previous study, western blotting identified that CD44 protein levels were remarkably increased in LNM-GC cells and their sEV, especially highly expressed in HGC-27L and HGC-27L-sEV but were hardly detectable in AGS and AGS-sEV (Additional file 7: Fig. S3A, B, Additional file 6: S2G, H). Endogenous CD44 protein levels declined obviously in BM-MSCs after treatment with the protein synthesis inhibitor CHX alone. CD44 protein was obviously increased in BM-MSCs treated with HGC-27L-sEV, but it was slightly decreased in presence of CHX (Additional file 7: Fig. S3C, Additional file 6: S2I). These data manifested that CD44 might be delivered to BM-MSCs through LNM-GC-sEV.

**Fig. 3:**
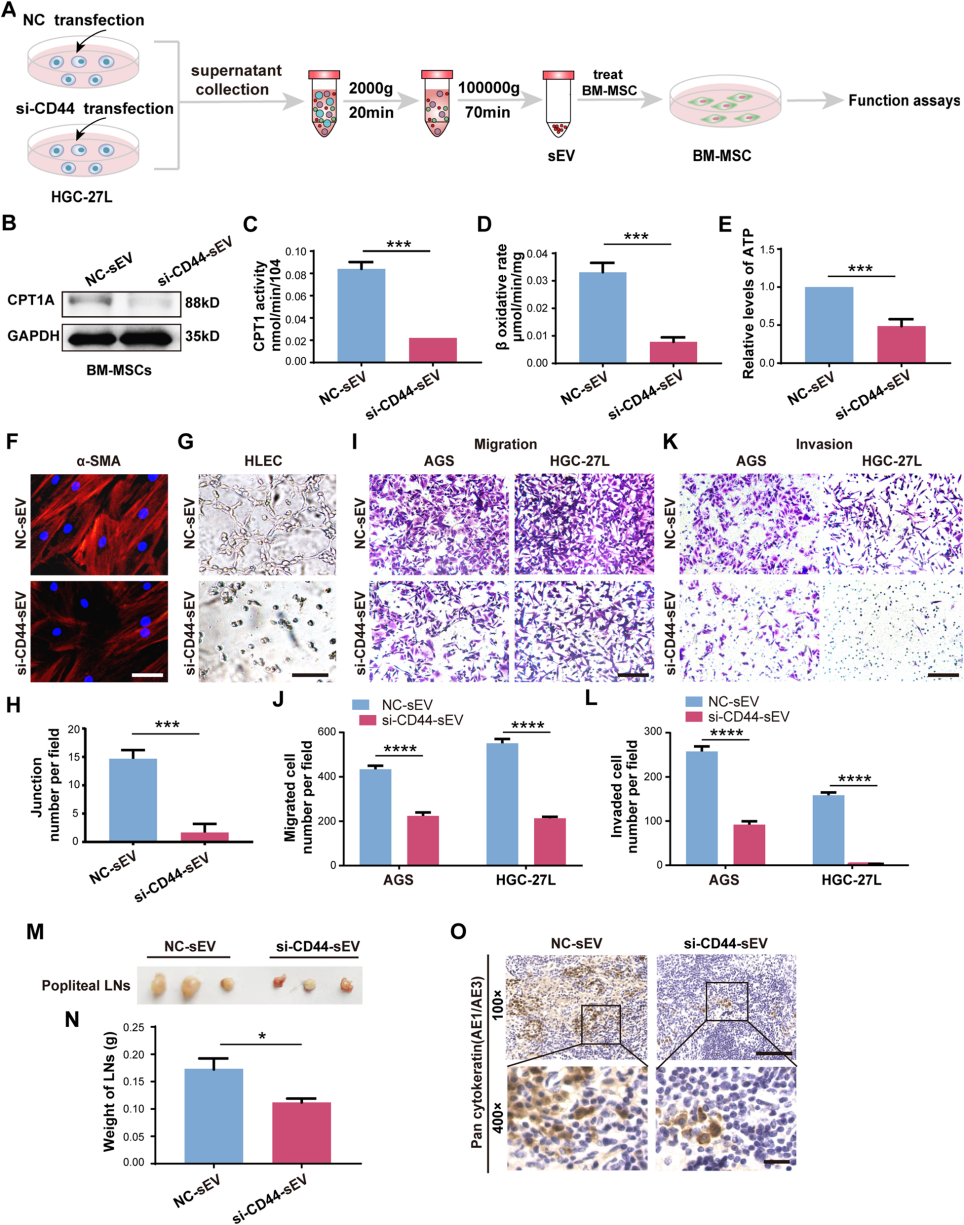
Knockdown of CD44 abrogates LNM-GC-sEV prompting FAO metabolic reprogramming of BM-MSCs. **A** Schematic illustration of BM-MSCs treated with sEV from NC and si-CD44 transfected HGC-27L respectively. **B** Western blotting analysis of CPT1A expression in the above sEV treated BM-MSCs. **C–E** Comparison of CPT1 activity, β-oxidation rate and ATP level in BM-MSCs between NC-sEV group and si-CD44-sEV group. **F–L** Comparison of BM-MSCs education by NC-sEV and si-CD44-sEV. **F** Immunofluorescence staining for α-SMA. **G, H** Tubule formation analysis. **I–L** Migration and invasion assays. **M** Representative images of popliteal LNs. **N** Weight of popliteal LNs. **O** Representative images of pan-cytokeratin (AE1/AE3) in popliteal LNs detection by IHC staining. *P < 0.05; ***P < 0.001; ****P < 0.0001

To explore the role of CD44-mediated BM-MSC education by LNM-GC-sEV, three siRNAs against CD44 were used to knock down CD44 content in HGC-27L-sEV (Additional file 7: Fig. S3D, E, Additional file 6: S2J, K). The most efficient siRNA #2 was used as si-CD44 to suppress CD44 in subsequent experiments. BM-MSCs were then treated with sEV isolated from HGC-27L transfected with NC and si-CD44, respectively (Fig. 3a). Compared to NC group, FAO activity in BM-MSCs were distinctly attenuated in si-CD44 group (Fig. 3b–e, Additional file 6: Fig. S2L). In vitro, α-SMA expression in BM-MSCs was significantly inhibited in si-CD44 group and their capabilities to promote HLEC tubule formation, GC migration and invasion were also impaired (Fig. 3f–l). In vivo, smaller and lighter popliteal lymph nodes and reduced staining areas of pan-cytokeratin AE1/AE3 were detected in the CD44-knockdown group (Fig. 3m–o). These results suggest that CD44 is pivotal for LNM-GC sEV to regulate FAO metabolic reprogramming of BM-MSCs.

### CD44 enrichment enables primary GC-sEV to acquire similar regulatory effects of LNM-GC-sEV on BM-MSCs

**Fig. 4:**
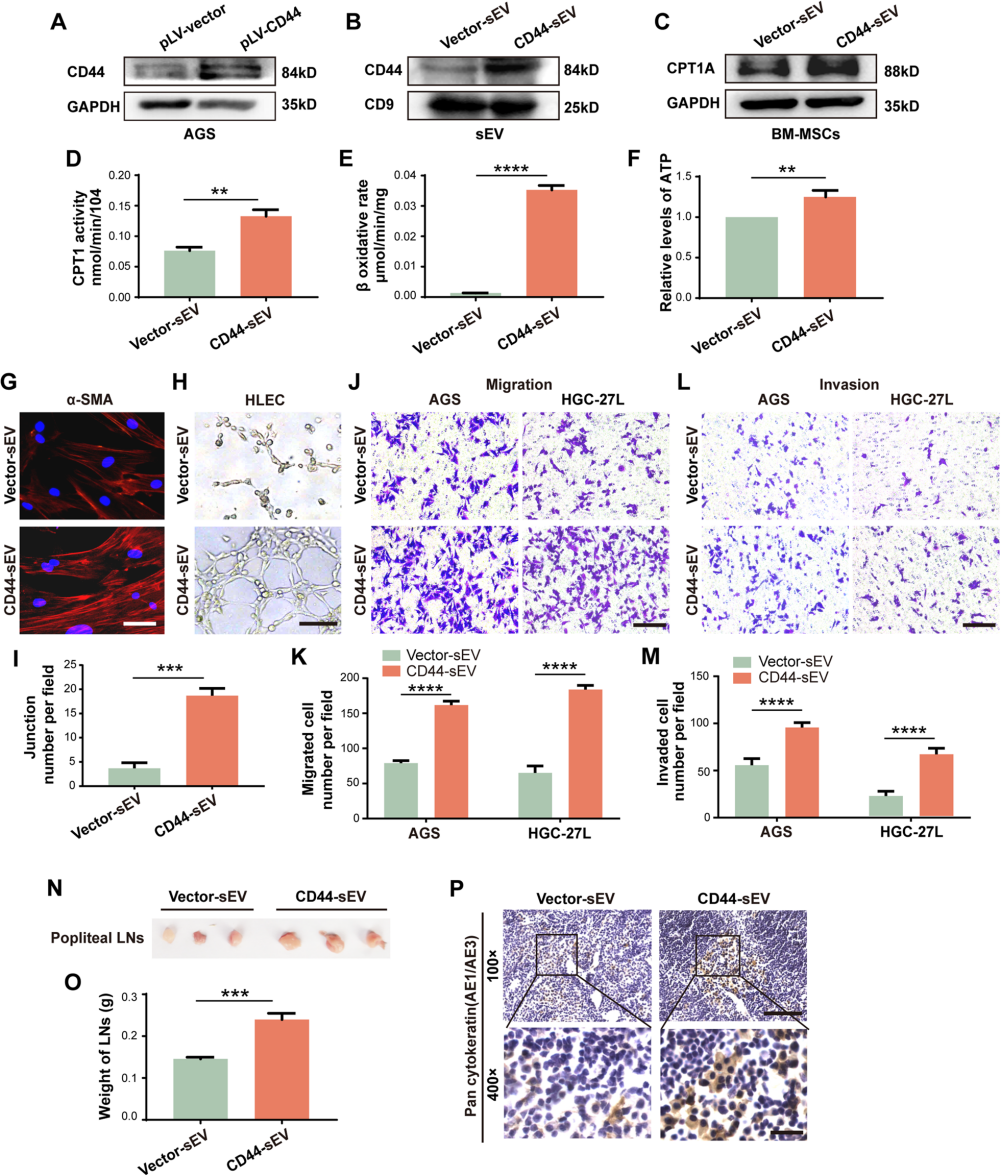
CD44 enrichment enables primary GC-sEV to acquire similar regulatory effects of LNM-GC-sEV on BM-MSCs. **A**, **B** Western blotting analysis of CD44 in AGS and their sEV after CD44 overexpression. **C** Western blotting analysis of CPT1A expression in BM-MSCs treated with vector-sEV and CD44-sEV. **D–F** Measurement of CPT1 activity, β-oxidation rate and ATP level in BM-MSCs treated with vector-sEV and CD44-sEV. **G–M** Comparison of BM-MSCs education by vector-sEV and CD44-sEV. **G** Immunofluorescence staining for α-SMA. **H**, **I** Tubule formation assay. **J–M** Transwell migration and invasion assays. **N** Representative images of popliteal LNs. **O** Popliteal LNs weight. **P** IHC staining of pan-cytokeratin (AE1/AE3) in popliteal LNs. **P < 0.01; ***P < 0.001; ****P < 0.0001

To further elucidate the crucial role of sEV-CD44 in modulating FAO metabolic reprogramming of BM-MSCs, CD44 was overexpressed in AGS using CD44-overexpressed lentivirus and CD44-enriched AGS-sEV was successfully obtained (Fig. 4a, b, Additional file 6: Fig. S2M, N). Corresponding vector lentivirus was used as a control. The above sEV derived from lentivirus infected AGS were used to treat BM-MSCs. Compared to the control group, FAO activity and α-SMA expression in BM-MSCs as well as the capabilities of BM-MSCs to promote HLEC tubule formation, GC migration and invasion were notably elevated and enhanced in CD44-enriched AGS-sEV group (Fig. 4c–m, Additional file 6: Fig. S2O), but these effects could be eliminated by etomoxir (Additional file 8: Fig. S4A–K, Additional file 6: S2P). In vivo, larger and heavier popliteal lymph nodes and increased positive staining area of pan-cytokeratin AE1/AE3 were observed in the CD44-enriched AGS-sEV group (Fig. 4n–p). In summary, CD44 was a critical protein cargo for LNM-GC-sEV modulating FAO metabolic reprogramming of BM-MSCs.

### ERK/PPARγ/CPT1A signaling mediates sEV-CD44 facilitating BM-MSC FAO

**Fig. 5:**
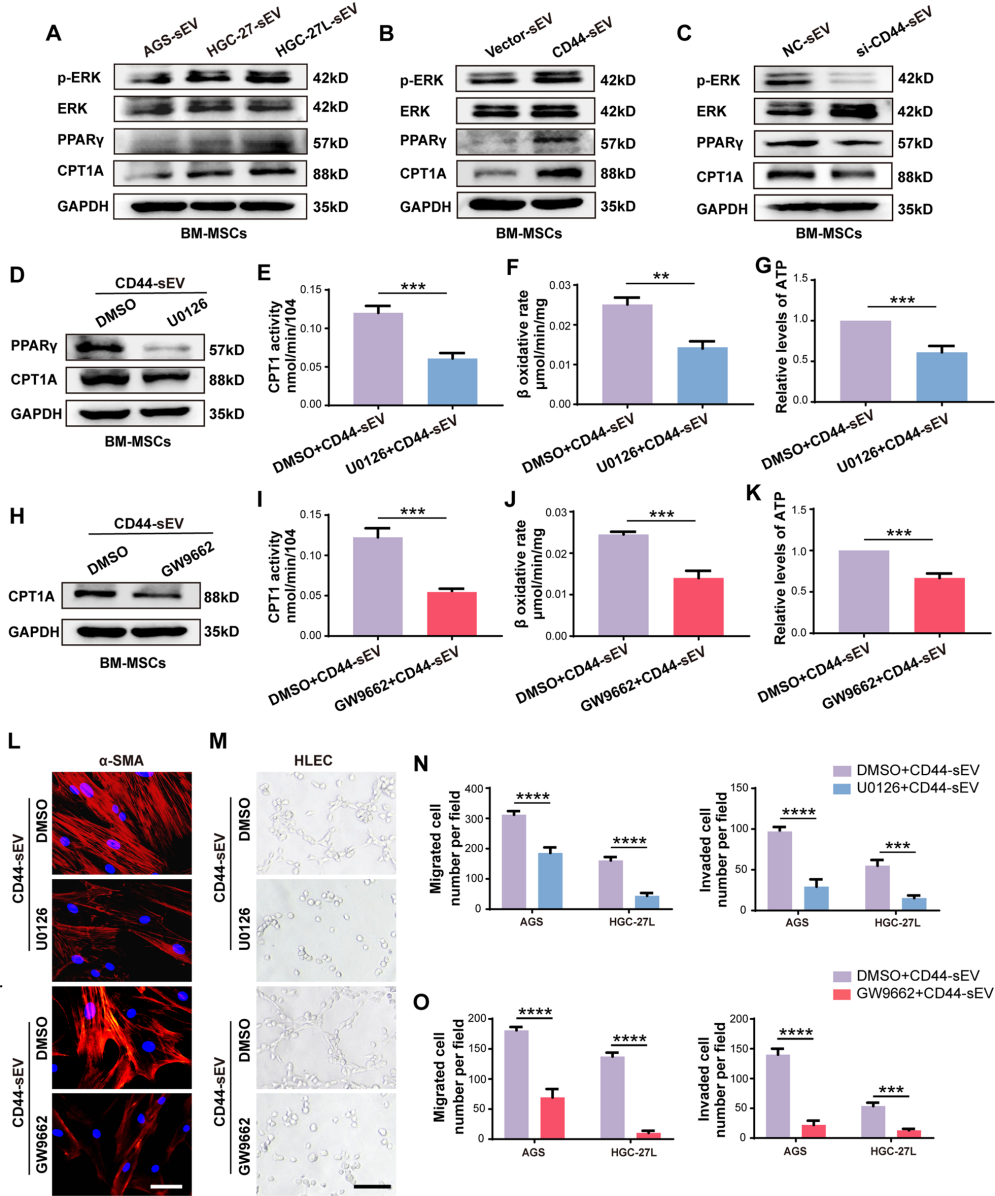
ERK/PPARγ/CPT1A signaling mediates sEV-CD44 facilitating BM-MSC FAO. **A–C** Western blotting analysis of P-ERK, ERK, PPARγ and CPT1A in BM-MSCs treated with GC-sEV, CD44-sEV and si-CD44-sEV. **D** Analysis of the effect of U0126 on PPARγ and CPT1A expression using Western blotting. **E–G** Analysis of the effect of U0126 on CPT1 activity, β-oxidation rate and ATP level in BM-MSCs. **H** Western blotting analysis of the effect of GW9662 on CPT1A protein expression. **I–K** The effect of GW9662 on CPT1 activity, β-oxidation rate, ATP level in BM-MSCs were analyzed. **L** α-SMA staining by immunofluorescence. **M** Tubule formation assay. **N**, **O** Migration and invasion analysis by transwell. **P < 0.01; ***P < 0.001; ****P < 0.0001

An important unanswered question is how sEV-CD44 potentiates FAO metabolic reprogramming of BM-MSCs. CPT1A is the rate-limiting enzyme of FAO and its protein levels in BM-MSCs was highly correlated with the content of CD44 in sEV (Figs. 3b, 4c), suggesting that sEV-CD44 may promote FAO through regulating CPT1A expression. It has been reported that PPARγ exerted a profound impact on FAO and functions as an upstream regulator of CPT1A [17, 18]. Other studies indicated that CD44 could activate ERK [19, 20], which was participated in PPARγ regulation [21, 22]. Therefore, we wondered whether ERK/PPARγ/CPT1A signaling pathway was involved in sEV-CD44-induced FAO metabolic reprogramming of BM-MSCs. As shown, the levels of phosphorylated ERK (p-ERK), PPARγ and CPT1A were markedly higher in BM-MSCs incubated with LNM-GC-sEV and CD44-enriched AGS-sEV than those treated with the corresponding control sEV (Fig. 5a, b, Additional file 6: Fig. S2Q, R). Conversely, the three proteins were all reduced in BM-MSCs treated with CD44-knockdown HGC-27L-sEV (Fig. 5c, Additional file 6: Fig. S2S). To clarify the functional roles of activated ERK and PPARγ during the process of BM-MSC education, we separately incubated BM-MSCs with ERK inhibitor U0126 and PPAR antagonist GW9662 before the indicated sEV treatment. U0126 significantly suppressed the elevation of PPARγ, CPT1A protein levels and FAO activity in BM-MSCs treated with CD44-enriched AGS-sEV (Fig. 5d–g, Additional file 6: Fig. S2T). GW9662 blocked CD44-enriched AGS-sEV inducing CPT1A expression and FAO activity in BM-MSCs (Fig. 5h–k, Additional file 6: Fig. S2U). Accordingly, α-SMA levels and tumor-promoting capacity of BM-MSCs enhanced by CD44-enriched AGS-sEV were obviously eliminated by U0126 and GW9662 (Fig. 5l–o, Additional file 9: Fig. S5A–F). These data indicate that sEV-CD44 modulates FAO metabolic reprogramming of BM-MSCs through ERK/PPARγ/CPT1A signaling pathway.

### *IL-8* and *STC1* are critical for the oncogenic roles of the educated BM-MSCs

**Fig. 6:**
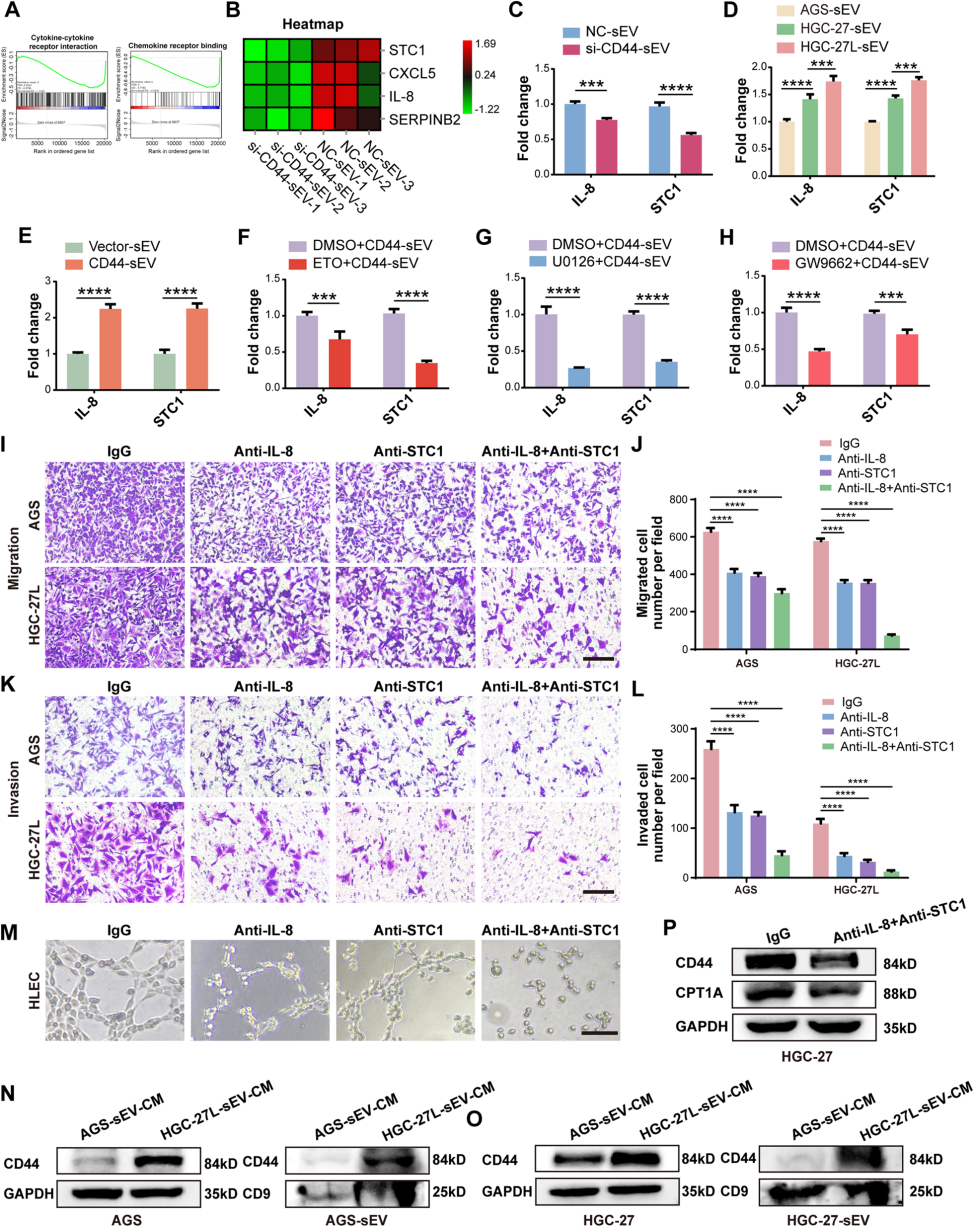
IL-8 and STC1 are critical for oncogenic roles of the educated BM-MSCs. **A** GSEA plots of indicated signature genes enriched in BM-MSCs incubated with si-CD44-sEV. **B** The Heatmap of the selected down-regulated genes related to secreted factors in si-CD44-sEV group compared with NC-sEV group.** C–H** *q*PCR of *IL-8* and *STC1* in BM-MSCs under indicated treatment. **I-L** Migration and invasion assays. **M** Tubule formation assay. **N**, **O** GC cells were treated with CM collected from BM-MSCs incubation with AGS-sEV and HGC-27L-sEV; Western blot analysis of CD44 levels in GC cells and their sEV. **P** CM from BM-MSCs educated by HGC-27L-sEV was incubated with IL-8 neutralizing antibody and STC1 antibody, respectively, and then were used to treat HGC-27. Western blotting analysis of CD44 and CPT1A in HGC-27. ***P < 0.001; ****P < 0.0001

The paracrine pathway is crucial for tumor-associated MSCs to promote tumor progression [23, 24]. To further investigate the potential paracrine signals for the oncogenic role of BM-MSCs educated by sEV-CD44, transcriptome sequencing was conducted to compare differential genes between NC-sEV-treated BM-MSCs and si-CD44-sEV-treated BM-MSCs. GSEA analysis showed that the differentially expressed genes between the two groups were closely related to cytokine-cytokine receptor interaction and Chemokine receptor binding (Fig. 6a). We focused on secretory factors and observed that Stanniocalcin 1 (*STC1*), C-X-C motif chemokine ligand 5 (*CXCL5*), C-X-C motif chemokine ligand 8 (*IL-8*) and *SERPINB2* were down-regulated in si-CD44-sEV group (Fig. 6b). Among these factors, *SERPINB2* expression is not significantly associated with clinicopathological features or prognosis of GC [25]. *STC1*, a glycoprotein hormone, is beneficial to the proliferation, invasion and metastasis of tumor cells [26]. It has been proved that the abnormal expression of *STC1* was significantly correlated with LNM of GC [27]. It is well known that *CXCL5* and *IL-8* are critical chemokines mediating GC metastasis [28, 29]. By analyzing the co-expression correlation between *CPT1A, IL-8, CXCL5, STC1* and *CD44* in stomach cancer tissues through TCGA database, we found that these genes except *CXCL5* were positively correlated with *CD44* and were positively associated with at least one MSC markers (*ENG, THY1, NT5E*) (Additional file 10: Fig. S6A–D). Therefore, *IL-8* and *STC1* were chosen for further functional verification. In accordance with sequencing data, silencing CD44 inhibited the mRNA expression of *IL-8* and *STC1* (Fig. 6c), while LNM-GC cells and CD44-enriched AGS-sEV up-regulated the mRNA expression of the two genes (Fig. 6d, e). Moreover, etomoxir, U0126 and GW9662 utilization obviously reduced the two genes expression in BM-MSCs, implying the link between FAO metabolic reprogramming and the two secretory factors (Fig. 6f–h).

To figure out the roles of *IL-8* and *STC1* for the educated BM-MSCs driving GC metastasis, neutralizing antibodies were separately or simultaneously used to block *IL-8* and *STC1* signaling of the BM-MSC-CM harvested after CD44-enriched AGS-sEV treatment. As shown, the capacities of the BM-MSC-CM to promote GC cells migration and invasion as well as HLEC tubule formation were partially impaired by anti-IL-8 or anti-STC1 alone, but were remarkably suppressed by combination of the two antibodies (Fig. 6i–m). Previously, we have demonstrated that CD44 was critical for GC cells lymphatic metastasis by promoting FAO metabolic reprogramming [11]. Therefore, we detected CD44 levels in GC cells and their sEVs after BM-MSC-CM treatment. As expected, both the cellular levels and sEV levels of CD44 were markedly increased by LNM-GC-sEV treated BM-MSC-CM (Fig. 6n, o, Additional file 6: Fig. S2V). Moreover, combination of anti-IL-8 and anti-STC1 pretreatment significantly abrogated the role of the educated BM-MSC-CM upregulating CD44 and CPT1A in GC cells (Fig. 6p, Additional file 6: Fig. S2W). These findings suggest that *IL-8* and *STC1* are responsible for the educated BM-MSC-CM to promote GC metastasis and a positive feedback regulatory loop possibly exists between GC cells and BM-MSCs.

### ATP might be the effective metabolite of FAO to facilitate BM-MSC education by activating STAT3 and NF-κB signaling

We next assessed whether FAO metabolite is favorable for educating BM-MSCs. ATP produced by FAO is essential for the biosynthesis of macromolecular substances and the proliferation of tumor cells. We used commercial ATP to treat BM-MSCs and observed that α-SMA expression and oncogenic function of BM-MSCs were enhanced by ATP (Additional file 10: Fig. S6E–K). Moreover, ATP elevated the expression of *IL-8* and *STC1* in BM-MSCs (Additional file 10: Fig. S6L). It has been revealed that extracellular ATP could induce STAT3 signaling and NF-κB pathway activation [30, 31], which links inflammation to cancer and is related to the release of some chemokines and STC1 [32–35]. Consistently, ATP stimulated the phosphorylation of STAT3 and p65 in BM-MSCs (Additional file 10: Figs. S6M, Additional file 6: S2X). In addition, LNM-GC-sEV and CD44-enriched AGS-sEV significantly enhanced the levels of phosphorylated STAT3 and p65 in BM-MSCs, while CD44-deficient sEV was just the opposite (Additional file 10: Fig. S6N, O, Additional file 6: S2Y, Z). Besides, etomoxir, U0126 and GW9662 treatment suppressed the phosphorylation of STAT3 and p65 caused by exosomal CD44 (Additional file 10: Fig. S6O, Additional file 6: S2Z). These results suggest that ATP might be the effective metabolite of FAO to facilitate BM-MSCs education by LNM-GC-sEV via activation of STAT3 and NF-κB, consequently promoting the release of crucial secretory factors involved in GC development.

### Increased expression of CD44, CPT1A, IL-8 and STC1 in GC tissues, serum and stroma are associated with poor prognosis of GC patients and LNM

**Fig. 7:**
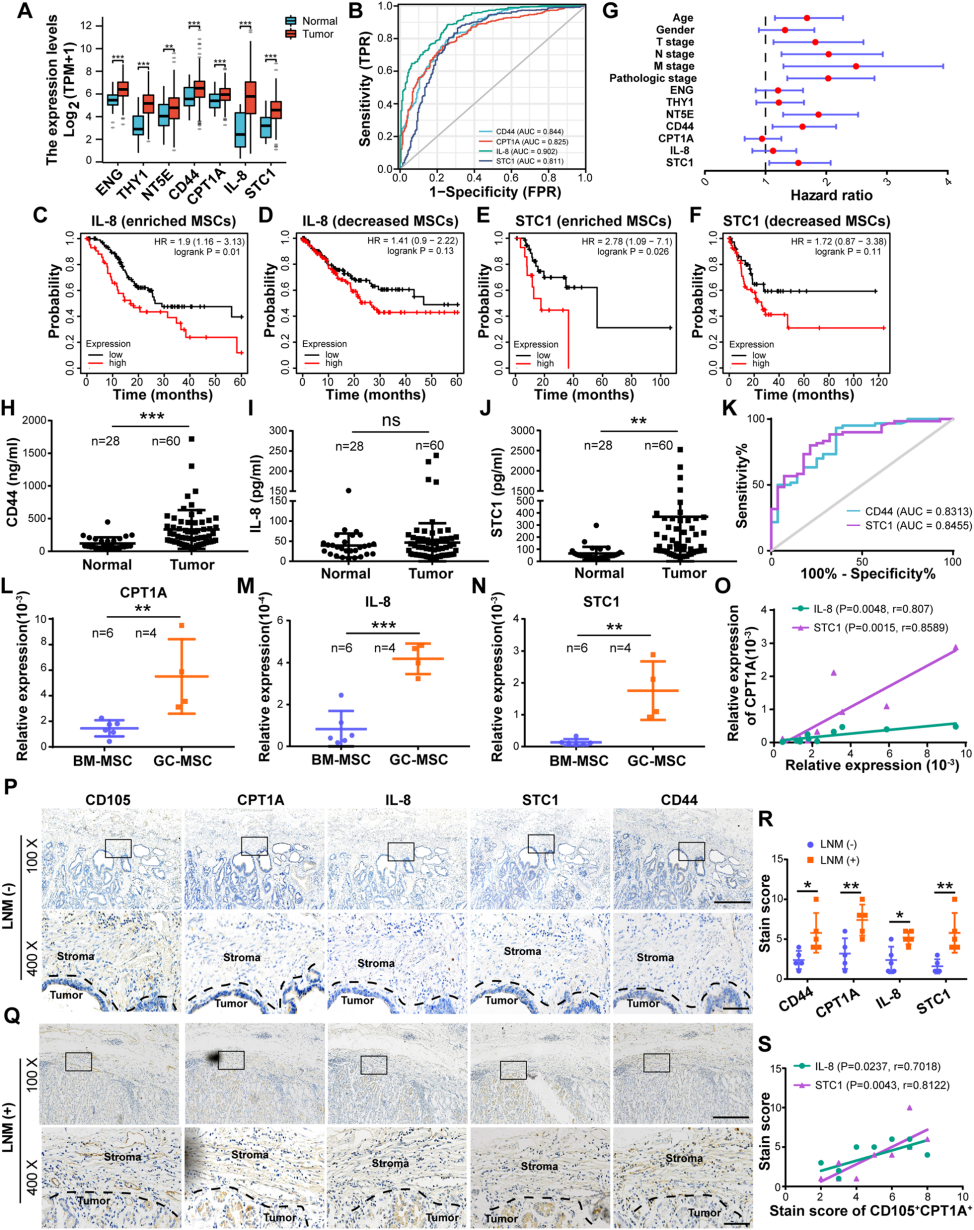
Increased expression of CD44, CPT1A, CXCL8 and STC1 in GC tissues, serum and stroma are associated with poor prognosis of GC patients and LNM. **A** The expression of *ENG, THY1, NT5E, CD44, CPT1A, IL-8* and *STC1* in TCGA GC tumor and GTEx normal stomach tissues. **B** Diagnostic value of *CD44, CPT1A, IL-8* and *STC1* for GC. **C–F** Kaplan–Meier analysis of *IL-8* and *STC1* association with OS of MSC enriched and decreased GC patients. **G** Forest plot of specific molecules effect on OS of GC patients. **H–J** Comparison of serum CD44, IL-8 and STC1 concentration between healthy people and GC patients. **K** Diagnostic value of serum CD44 and STC1 for GC. **L–N** The mRNA levels of *IL-8* and *STC1* in BM-MSCs and GC-MSCs were detected by RT-*q*PCR. **O** Correlation of *CPT1A* with *IL-8* and *STC1* in MSCs. **P–S** IHC staining analysis of primary tumor sites in GC patients with or without LNM. **P**, **Q** Representative images. **R** IHC staining scores of CD44, CPT1A, IL-8, and STC1 in GC stroma (n = 5 samples for each group). **S** Correlation of CPT1A with IL-8 and STC1 in CD105 positive stromal cells. Data are shown as the mean ± SEM. Non-significant (ns) *P* > 0.05; *P < 0.05; **P < 0.01; ***P < 0.001

We have confirmed that *CD44*, *CPT1A*, *IL-8* and *STC1* were key molecules involved in the FAO metabolic reprogramming of BM-MSCs. To explore the expression profile of these molecules and their prognostic values in GC, TCGA data was used to compare their levels between normal tissues and GC tissues. Three MSC markers including *ENG*, *THY1* and *NT5E* were also included for analysis. As shown, all these selected molecules were significantly increased in GC tissues (Fig. 7a). ROC curves established for *CD44*, *CPT1A*, *IL-8* and *STC1* showed that the four genes had superior diagnostic performance for GC (Fig. 7b). Our previous study revealed that GC patients with higher levels of *CD44* and *CPT1A* had shortened survivals based on Kaplan Meier plotter database analysis [11]. *ENG*, *THY1*, *NT5E*, *IL-8* and *STC1* presented similar correlations with the OS of GC patients (Additional file 11: Fig. S7A–E). Furthermore, the prognostic values of the above molecules were further analyzed based on MSC abundance in GC tissues. As shown, high levels of *IL-8* and *STC1* were correlated with shortened OS in the MSC-enriched group, not in the MSC-reduced group (Fig. 7c–f). Cox regression analysis and identified that *NT5E*, *CD44* and *STC1* were associated with OS, among which *NT5E* was an independent prognostic factor (Fig. 7g, Additional file 11: Fig. S7F, Additional file 3: Table S3), and that *NT5E*, *CD44*, and *CPT1A* were correlated with the progression free survival (PFS), among which *CD44* was an independent prognostic indicator (Additional file 11: Fig. S7G, H, Additional file 4: Table S4). Besides, the serum levels of CD44, IL-8 and STC1 were compared between healthy people and GC patients by ELISA. CD44 and STC1, not IL-8 levels were observed to be higher in GC patients than those in healthy people, and had good performance for GC diagnosis (Fig. 7h–k). These findings suggest that CD44, CPT1A, IL-8 and STC1 were abnormally increased in GC tissues and serum, and may be explored as diagnostic and prognostic indicators for GC patients.

To further confirm the expression levels of *CPT1A*, *IL-8* and *STC1* altered in GC-MSCs, six BM-MSC cell lines and four GC-MSC cell lines were randomly chosen for *q*PCR analysis. Compared with BM-MSCs, their expression levels were all increased in GC-MSCs (Fig. 7l–n). Moreover, *IL-8* and *STC1* levels exhibited a positive correlation with *CPT1A* levels in MSCs, respectively (Fig. 7o). To further analyze whether CD44, CPT1A, IL-8 and STC1 expression levels in tumor stroma were correlated with lymphatic metastasis, IHC was conducted to measure their levels in GC tissues from five patients with and without LNM. CD105 was used as a marker for GC associated stromal cells. The positive rates of CD44, CPT1A, IL-8 and STC1 in stromal cells were higher in patients with LNM than those without LNM (Fig. 7p–r). Moreover, the staining scores of IL-8 and STC1 were positively correlated with CPT1A staining scores in CD105 positive stromal cells (Fig. 7s). These data indicate that CD44, CPT1A, IL-8 and STC1 were upregulated in GC associated MSCs and correlated with LNM of GC patients.

## Discussion

**Fig. 8:**
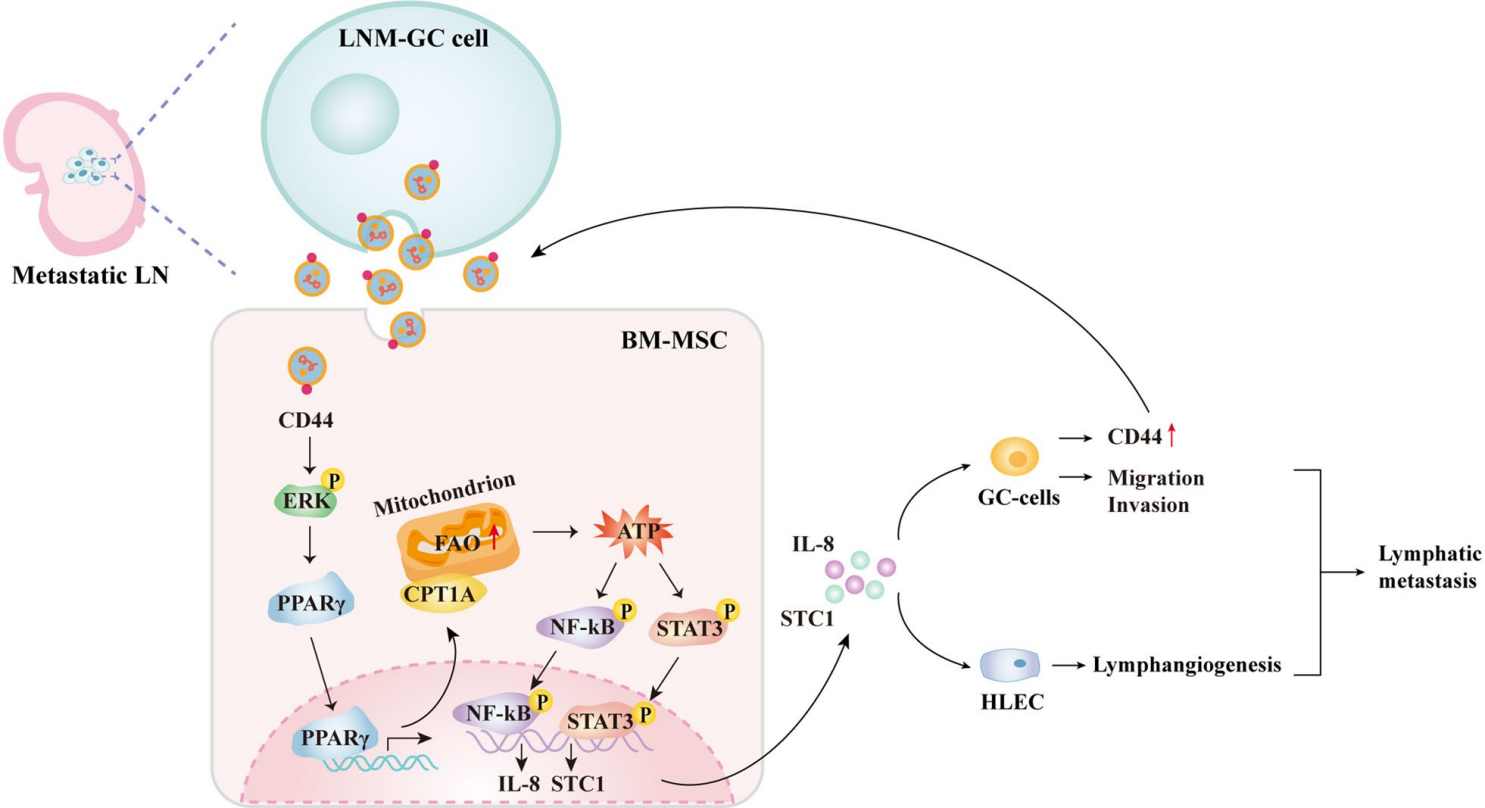
Schematic diagram of LNM-GC cell sEV-CD44 induces FAO metabolic reprogramming of BM-MSCs via ERK-PPARγ-CPT1A signaling to stimulate the release of IL-8 and STC1 and promote GC lymphatic metastasis

LNM is a common route of GC metastasis and more than 50% of GC patients have LNM at initial diagnosis or surgical resection [36]. Identifying the mechanism of LNM is important for accurate diagnosis and appropriate surgical management of GC patients. In this study, we revealed a novel mechanism connecting FAO metabolic reprogramming of BM-MSCs to LNM of GC. We found that sEV-CD44 derived from LNM-GC cells increased FAO activity in BM-MSCs through activating ERK/PPARγ/CPT1A signaling pathway, thereby leading to IL-8 and STC1 secretion to facilitate LNM by enhancing GC cells migration, invasion and lymphangiogenesis as well as inducing cellular and sEV CD44 expression to form positive feedback loop between GC cells and BM-MSCs (Fig. 8).

It is well-known that BM-MSCs are an important origin of tumor microenvironment cells and play a pivotal role in tumor metastasis [37]. Accumulating evidence proved that sEV mediates cellular communication between tumor cells and stromal cells during tumor progression. In particular, tumor cell-derived sEV could induce trans-differentiation of BM-MSCs into different tumor stromal cells, thus providing an in vitro cellular model for elucidating the underlying mechanisms [6, 7]. Previously, when we established BM-MSCs trans-differentiation models using different GC-sEV, we accidentally found that LNM-GC cells originated sEV was able to educate BM-MSCs compared to primary GC cells [8]. To further make clear whether the capacity to educate BM-MSCs was correlated with LNM abilities of GC cells, in the present work, we established a highly metastatic GC cell line HGC-27L by serial transplantation of parental HGC-27 in vivo and found that HGC-27L-sEV was more capable to educate BM-MSCs than the parental HGC-27-sEV. The findings implied that with the enhancement of the LNM ability of GC cells, their capacity to educate BM-MSCs was also enhanced. Similarly, it has been reported that sEV derived from high-metastatic colorectal cancer (CRC) cells is more likely to induce CAF activation than that from low-metastatic CRC cells [16]. Furthermore, tracking of MSCs in the mouse model showed that MSCs tend to home to the metastatic site rather than the primary tumor [38]. These studies further support our findings and indicate that metastatic tumor cells possess an enhanced capability to educate stromal cells to create a favorable microenvironment for tumor cell metastasis. Previously, we also successfully isolated MSC-like cells from the regional metastatic LNs of GC [8]. Meanwhile, MSCs were shown to be involved in LNM of GC [39]. Taken together, these findings suggest that MSCs are closely related to the lymphatic metastasis of GC. Therefore, it is urgent and necessary to interpret the mechanism of the lymphatic metastasis of GC from the perspective of educated BM-MSCs.

Metabolic reprogramming is a hall marker of tumors. Extensive studies revealed that stromal cells underwent metabolic reprogramming to create favorable microenvironment for tumor metastasis. FAO is a primary source for energy production [40]. With the increasing importance of FAO in tumor development, alterations in fatty acid metabolism have been highlighted. Our previous studies uncovered that FAO activity was increased in GC-MSCs and indispensable for GC-MSCs to prompt GC progression. Since LNM-GC-sEV could efficiently induce trans-differentiation of BM-MSCs into GC-MSC-like cells, we speculated that FAO metabolic reprogramming was more probably involved in the process of BM-MSCs education. As expected, the educated BM-MSCs displayed increased FAO activity and FAO blockade by etomoxir significantly eliminated the role of LNM-GC-sEV. These findings indicate that FAO metabolic reprogramming is critical for LNM-GC-sEV inducing BM-MSCs to acquire tumor-promoting phenotype and function. Through transcriptome sequencing and interference of secretory factors, we further provided evidence that ATP might be the effective metabolite to elicit the educated BM-MSCs secretion of IL-8 and STC1 to promote GC cells migration, invasion and lymphangiogenesis by activating STAT3 and NF-κB. However, we must be aware that ATP is not a unique metabolite of FAO and may come from other metabolic pathways. Combined with the findings in the present study, we inferred that ATP might be mainly produced by FAO. Whether other metabolic pathways are involved in this process requires further comprehensive evaluation by metabolomics and other methods, but our results at least demonstrate the regulatory role of ATP in BM-MSCs education.sEV is known to carry a variety of bioactive molecules while proteins are the most abundant components. Multiple studies have shown that sEV secreted by metastatic tumor cells is rich in metastasis-promoting proteins [41, 42], and that prompts us to investigate which proteins in sEV mediate BM-MSCs education. Formerly, we compared protein differences between LNM-GC-sEV and primary GC-sEV by label-free quantitation detection, and identified high accumulation of CD44 in LNM-GC-sEV. CD44 levels in cells and sEV were highly correlated with the ability of the lymphatic metastasis of GC. Moreover, sEV-CD44 regulated FAO-mediated malignant phenotype transmission [11]. Based on these, we decided to investigate whether CD44 in sEV educated BM-MSCs by regulating FAO. The PPARs family is regarded as fatty acid sensors regulating metabolism, in which PPARα and PPARγ regulate the transcriptional expression of CPT1A [17, 43]. In this study, we found that the expression of PPARγ and CPT1A was highly correlated with each other. However, the relationship between CD44 and PPARγ has not been reported. Several studies suggest that CD44 is a common upstream regulator of the ERK pathway [19, 20]. In consequence, ERK and PPARγ were selected as linking molecules to investigate the regulation of CPT1A mediated by CD44. Subsequent experiments confirmed that CD44 in sEV triggered FAO-dependent reprogramming of BM-MSCs via ERK/PPARγ/CPT1A signaling. However, the specific regulation between these molecules has not been revealed in our study and needs to be clarified in future studies.

In order to comprehensively identify the clinical significance of key molecules involved in FAO metabolic reprogramming of BM-MSCs by sEV-CD44, we analyzed their levels in GC tissues, sera and GC associated stroma. All of these molecules were highly expressed in GC tissues and associated with poor prognosis of GC patients. Serum levels of CD44 and STC1 were increased in healthy people compared to GC patients. But the serum levels of IL-8 didn't show any differences, which is contrary to the findings of other studies [44, 45]. This discrepancy may be caused by sampling errors. In agreement with the recent study [46], three MSC markers were all increased in GC tissues and abundant MSCs were associated with poor prognosis of GC patients, which confirmed the important role of MSCs in the progression of GC. Given the complexity of GC tissues and the lack of specificity of markers, we compared the expression and correlation of CPT1A, IL-8 and STC1 in BM-MSCs and GC-MSCs. These molecules were remarkably up-regulated in GC-MSCs. Moreover, CD105 was used as a marker of MSCs in GC stroma and following IHC assay revealed that these molecules were increased in GC associated MSCs and highly correlated with LNM. Based on the clinical sample analysis, the key molecules mediated BM-MSCs education are expected to be explored as molecular targets for the diagnosis, prognosis and treatment of GC. The potential of these molecules in clinical applications needs to be further comprehensively evaluated in expanded samples.

## Supplementary Information

**Additional file 1. Table S1:** Oligonucleotide sequences.

**Additional file 2. Table S2:** Primer sequences.

**Additional file 3. Table S3:** Univariate and multivariate Cox regression of overall survival.

**Additional file 4. Table S4:** Univariate and multivariate Cox regression of progression free survival.

**Additional file 5. Fig. S1:** Characterization of sEV by TEM, Western blot analysis and NTA. A Representative images under transmission electron microscopy. B Particle size and distribution detected by nanoparticle tracking analysis. C Western blotting analysis for sEV marker CD63 and CD9.

**Additional file 6. Fig. S2:** LNM-GC-sEV increases FAO activity in BM-MSCs. A CPT1A expression in BM-MSCs treated with sEV derived from GC cells with different LNM capacity was measured by Western Blot. B–D The effect of GC-sEV on the FAO activity in BM-MSCs was evaluated, including activity of CPT1, β-oxidation rate and ATP level. E–Z Relative levels of protein. *P < 0.05; **P < 0.01; ***P < 0.001; ****P < 0.0001.

** Additional file 7. Fig. S3:** CD44 is highly expressed in LNM-GCs and their sEV. A, B Comparison of cellular and sEV levels of the CD44 protein in GC cells by western blotting. C Effect of CHX on CD44 protein levels in BM-MSCs treated with or without HGC-27L-sEV. D Screening for the most efficient siRNA against CD44 in HGC-27L. E Western blotting analysis of CD44 contents in sEV derived from HGC-27L after transfection with siRNA and NC.

**Additional file 8. Fig. S4:** Enhanced FAO is required for the education of BM-MSCs mediated by CD44. A Analysis of the effect of etomoxir on CPT1A expression in BM-MSCs treated with CD44-sEV using Western blotting. B–D The activity of CPT1, β-oxidation rate and ATP level detection in BM-MSCs. E Immunofluorescence staining for α-SMA. F, G Tubule formation assay. H–K Migration and invasion assays. ***P < 0.001; ****P < 0.0001.

**Additional file 9. Fig. S5:** U0126 and GW9662 suppress tumor-promoting capacity of BM-MSCs. A, B Quantification of formed tubule junctions. C–F Morphology of migrated and invaded cells. ***P < 0.001.

**Additional file 10. Fig. S6:** ATP might be the effective metabolite of FAO to facilitate BM-MSCs education by activating STAT3 and NF-κB signaling. A Correlation of CD44 with CPT1A, IL-8, CXCL5 and STC1 were analyzed according to the data of TCGA-STAD. B–D Correlation of MSC markers ENG, THY1 and NT5E with CD44, CPT1A, IL-8, CXCL5 and STC1 in GC tissue from data of TCGA-STAD. E–K Effect of ATP on the education of BM-MSCs. E Immunofluorescence staining for α-SMA. F, G Tubule formation assay. H–K Migration and invasion assays. L The mRNA levels of IL-8 and STC1 in BM-MSCs with or without ATP were measured by RT-qPCR. M–O Western blotting analysis of p-STAT3 and p-P65 in BM-MSCs under indicated treatment. **P < 0.01; ****P < 0.0001.

**Additional file 11. Fig. S7:** High expression of MSC markers, CD44, CPT1A, CXCL8 and STC1 predict poor survival of GC. A–E Kaplan–Meier analysis of ENG, THY1, NT5E, IL-8 and STC1 association with OS of GC patients. F Forest plot of specific molecules effect on OS of GC patients. G, H Forest plot of specific molecules effect on FPS of GC patients.

## Data Availability

The datasets used in this study are available from the corresponding author on reasonable request.
