# Supplementary material for: Gastric cancer cell-originated small extracellular vesicle induces metabolic reprogramming of BM-MSCs through ERK-PPARγ-CPT1A signaling to potentiate lymphatic metastasis
Source: Cancer Cell Int. 2023 May 9;23:87. doi: 10.1186/s12935-023-02935-5 (PMC10169337; doi:10.1186/s12935-023-02935-5)
Supplement: Supplementary file 2 — Additional file 2. Table S2: Primer sequences. [file 12935_2023_2935_MOESM2_ESM.docx]

**Table S2 Primer sequences**

| **Primers** | **Sequences (5’-3’)** |
| --- | --- |
| β-actin | F:CACGAAACTACCTTCAACTCC  R:CATACTCCTGCTTGCTGATC |
| IL-8 | F:GCTCTGTGTGAAGGTGCAGTTT  R:TTCTGTGTTGGCGCAGTGT |
| STC1 | F:TTCGTTGCCTCAACAGTGCT  R:TGACGAATGCTTTTCCCTGAGT |
