# Supplementary material for: Gastric cancer cell-originated small extracellular vesicle induces metabolic reprogramming of BM-MSCs through ERK-PPARγ-CPT1A signaling to potentiate lymphatic metastasis
Source: Cancer Cell Int. 2023 May 9;23:87. doi: 10.1186/s12935-023-02935-5 (PMC10169337; doi:10.1186/s12935-023-02935-5)
Supplement: Supplementary file 3 — Additional file 3. Table S3: Univariate and multivariate Cox regression of overall survival. [file 12935_2023_2935_MOESM3_ESM.docx]

**Table S3 Univariate and multivariate Cox regression of overall survival**

| Characteristics | Total(N) | Univariate analysis | |  | Multivariate analysis | |
| --- | --- | --- | --- | --- | --- | --- |
|  |  | Hazard ratio (95% CI) | P value |  | Hazard ratio (95% CI) | P value |
| Age | 367 |  |  |  |  |  |
| <=65 | 163 | Reference |  |  |  |  |
| >65 | 204 | 1.620 (1.154-2.276) | **0.005** |  | 1.823 (1.237-2.687) | **0.002** |
| Gender | 370 |  |  |  |  |  |
| Female | 133 | Reference |  |  |  |  |
| Male | 237 | 1.267 (0.891-1.804) | 0.188 |  | 1.658 (1.113-2.470) | **0.013** |
| Histological type | 369 |  |  |  |  |  |
| Diffuse Type | 63 | Reference |  |  |  |  |
| Mucinous Type&Papillary Type&Signet Ring Type&Tubular Type | 104 | 0.932 (0.564-1.539) | 0.783 |  | 0.769 (0.437-1.352) | 0.361 |
| Not Otherwise Specified | 202 | 1.175 (0.748-1.844) | 0.485 |  | 1.128 (0.679-1.871) | 0.642 |
| T stage | 362 |  |  |  |  |  |
| T1&T2 | 96 | Reference |  |  |  |  |
| T3&T4 | 266 | 1.719 (1.131-2.612) | **0.011** |  | 1.130 (0.660-1.936) | 0.655 |
| N stage | 352 |  |  |  |  |  |
| N0 | 107 | Reference |  |  |  |  |
| N1&N2&N3 | 245 | 1.925 (1.264-2.931) | **0.002** |  | 1.602 (0.877-2.927) | 0.125 |
| M stage | 352 |  |  |  |  |  |
| M0 | 327 | Reference |  |  |  |  |
| M1 | 25 | 2.254 (1.295-3.924) | **0.004** |  | 2.507 (1.336-4.703) | **0.004** |
| Pathologic stage | 347 |  |  |  |  |  |
| Stage I&Stage II | 160 | Reference |  |  |  |  |
| Stage III&Stage IV | 187 | 1.947 (1.358-2.793) | **<0.001** |  | 1.228 (0.698-2.161) | 0.477 |
| ENG | 370 |  |  |  |  |  |
| Low | 186 | Reference |  |  |  |  |
| High | 184 | 1.164 (0.838-1.618) | 0.364 |  | 0.857 (0.564-1.303) | 0.471 |
| THY1 | 370 |  |  |  |  |  |
| Low | 185 | Reference |  |  |  |  |
| High | 185 | 1.177 (0.848-1.634) | 0.330 |  | 1.166 (0.770-1.766) | 0.469 |
| NT5E | 370 |  |  |  |  |  |
| Low | 184 | Reference |  |  |  |  |
| High | 186 | 1.805 (1.290-2.524) | **<0.001** |  | 1.874 (1.272-2.761) | **0.001** |
| CD44 | 370 |  |  |  |  |  |
| Low | 185 | Reference |  |  |  |  |
| High | 185 | 1.553 (1.115-2.162) | **0.009** |  | 1.379 (0.946-2.011) | 0.095 |
| CPT1A | 370 |  |  |  |  |  |
| Low | 186 | Reference |  |  |  |  |
| High | 184 | 0.910 (0.656-1.262) | 0.573 |  | 0.885 (0.621-1.260) | 0.497 |
| CXCL8 | 370 |  |  |  |  |  |
| Low | 185 | Reference |  |  |  |  |
| High | 185 | 1.086 (0.783-1.507) | 0.620 |  | 0.868 (0.599-1.259) | 0.456 |
| STC1 | 370 |  |  |  |  |  |
| Low | 184 | Reference |  |  |  |  |
| High | 186 | 1.484 (1.062-2.074) | **0.021** |  | 1.058 (0.706-1.586) | 0.784 |
