# Supplementary material for: Gastric cancer cell-originated small extracellular vesicle induces metabolic reprogramming of BM-MSCs through ERK-PPARγ-CPT1A signaling to potentiate lymphatic metastasis
Source: Cancer Cell Int. 2023 May 9;23:87. doi: 10.1186/s12935-023-02935-5 (PMC10169337; doi:10.1186/s12935-023-02935-5)
Supplement: Supplementary file 4 — Additional file 4. Table S4: Univariate and multivariate Cox regression of progression free survival. [file 12935_2023_2935_MOESM4_ESM.docx]

**Table S4 Univariate and multivariate Cox regression of progression free survival**

| Characteristics | Total(N) | Univariate analysis | |  | Multivariate analysis | |
| --- | --- | --- | --- | --- | --- | --- |
|  |  | Hazard ratio (95% CI) | P value |  | Hazard ratio (95% CI) | P value |
| Age | 369 |  |  |  |  |  |
| <=65 | 164 | Reference |  |  |  |  |
| >65 | 205 | 0.858 (0.603-1.221) | 0.395 |  | 1.133 (0.760-1.687) | 0.540 |
| Gender | 372 |  |  |  |  |  |
| Female | 133 | Reference |  |  |  |  |
| Male | 239 | 1.638 (1.099-2.440) | **0.015** |  | 2.113 (1.366-3.269) | **<0.001** |
| Histological type | 371 |  |  |  |  |  |
| Diffuse Type | 63 | Reference |  |  |  |  |
| Mucinous Type&Papillary Type&Signet Ring Type&Tubular Type | 104 | 0.775 (0.472-1.275) | 0.316 |  | 0.820 (0.456-1.475) | 0.508 |
| Not Otherwise Specified | 204 | 0.793 (0.503-1.251) | 0.319 |  | 1.055 (0.624-1.784) | 0.842 |
| T stage | 364 |  |  |  |  |  |
| T1&T2 | 97 | Reference |  |  |  |  |
| T3&T4 | 267 | 1.705 (1.095-2.654) | **0.018** |  | 1.337 (0.766-2.336) | 0.307 |
| N stage | 354 |  |  |  |  |  |
| N0 | 108 | Reference |  |  |  |  |
| N1&N2&N3 | 246 | 1.640 (1.075-2.501) | **0.022** |  | 1.400 (0.778-2.520) | 0.262 |
| M stage | 353 |  |  |  |  |  |
| M0 | 328 | Reference |  |  |  |  |
| M1 | 25 | 2.224 (1.194-4.144) | **0.012** |  | 2.316 (1.139-4.710) | **0.020** |
| Pathologic stage | 349 |  |  |  |  |  |
| Stage I&Stage II | 161 | Reference |  |  |  |  |
| Stage III&Stage IV | 188 | 1.676 (1.154-2.435) | **0.007** |  | 1.005 (0.571-1.769) | 0.986 |
| ENG | 372 |  |  |  |  |  |
| Low | 186 | Reference |  |  |  |  |
| High | 186 | 1.065 (0.748-1.516) | 0.727 |  | 0.991 (0.663-1.482) | 0.967 |
| THY1 | 372 |  |  |  |  |  |
| Low | 186 | Reference |  |  |  |  |
| High | 186 | 0.958 (0.673-1.363) | 0.811 |  |  |  |
| NT5E | 372 |  |  |  |  |  |
| Low | 186 | Reference |  |  |  |  |
| High | 186 | 1.412 (0.990-2.014) | 0.057 |  | 1.549 (1.035-2.319) | **0.034** |
| CD44 | 372 |  |  |  |  |  |
| Low | 186 | Reference |  |  |  |  |
| High | 186 | 1.522 (1.066-2.175) | **0.021** |  | 1.563 (1.051-2.324) | **0.027** |
| CPT1A | 372 |  |  |  |  |  |
| Low | 187 | Reference |  |  |  |  |
| High | 185 | 0.702 (0.493-1.002) | 0.051 |  | 0.630 (0.430-0.924) | **0.018** |
| CXCL8 | 372 |  |  |  |  |  |
| Low | 187 | Reference |  |  |  |  |
| High | 185 | 0.739 (0.518-1.055) | 0.096 |  | 0.762 (0.514-1.131) | 0.178 |
| STC1 | 372 |  |  |  |  |  |
| Low | 186 | Reference |  |  |  |  |
| High | 186 | 1.163 (0.816-1.657) | 0.403 |  | 0.982 (0.645-1.496) | 0.932 |
