# Supplementary figures and images for: Gastric cancer cell-originated small extracellular vesicle induces metabolic reprogramming of BM-MSCs through ERK-PPARγ-CPT1A signaling to potentiate lymphatic metastasis
Source: Cancer Cell Int. 2023 May 9;23:87. doi: 10.1186/s12935-023-02935-5 (PMC10169337; doi:10.1186/s12935-023-02935-5)

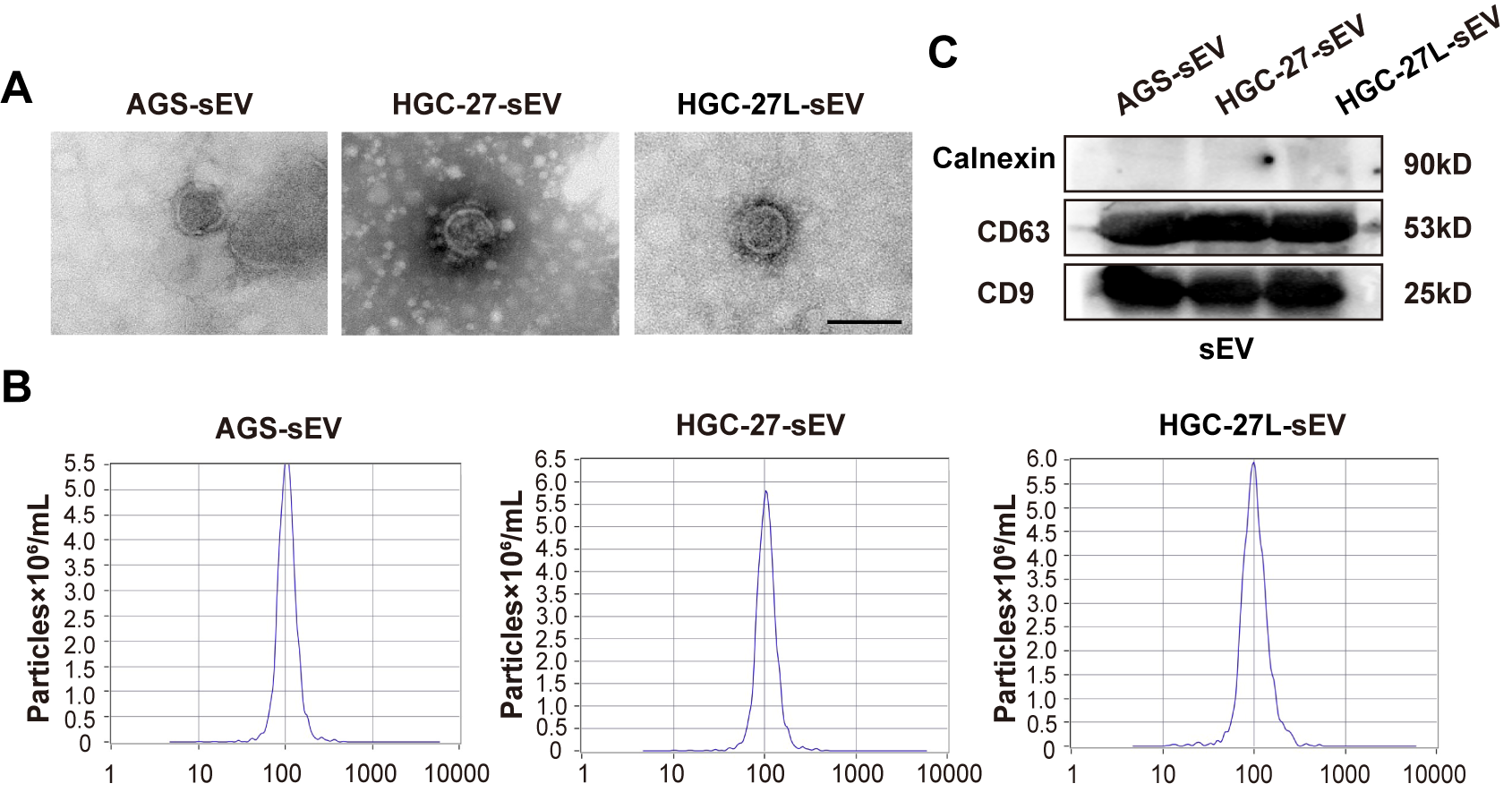

Supplement: Supplementary file 5 — Additional file 5. Fig. S1: Characterization of sEV by TEM, Western blot analysis and NTA. A Representative images under transmission electron microscopy. B Particle size and distribution detected by nanoparticle tracking analysis. C Western blotting analysis for sEV marker CD63 and CD9. [file 12935_2023_2935_MOESM5_ESM.tif]

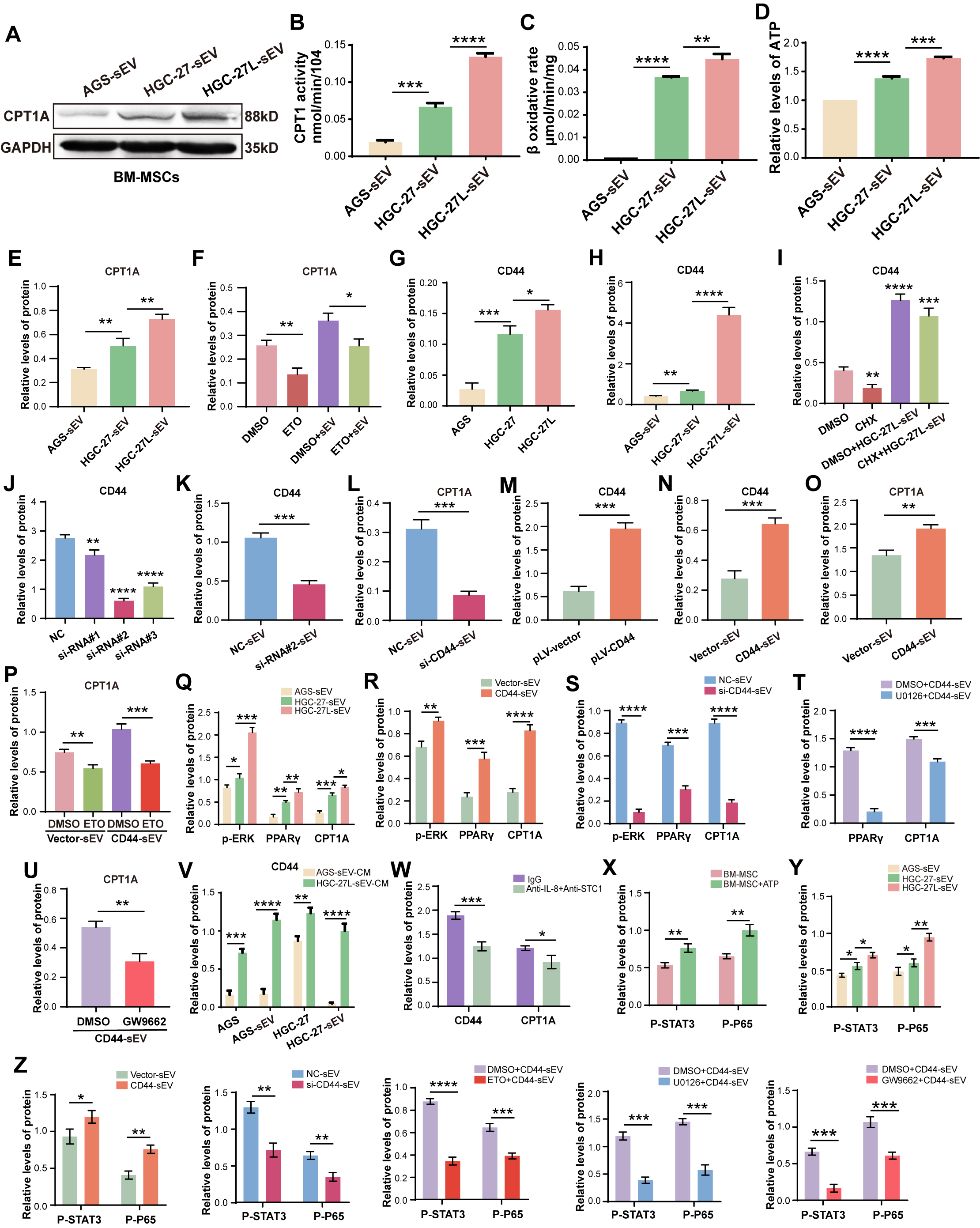

Supplement: Supplementary file 6 — Additional file 6. Fig. S2: LNM-GC-sEV increases FAO activity in BM-MSCs. A CPT1A expression in BM-MSCs treated with sEV derived from GC cells with different LNM capacity was measured by Western Blot. B–D The effect of GC-sEV on the FAO activity in BM-MSCs was evaluated, including activity of CPT1, β-oxidation rate and ATP level. E–Z Relative levels of protein. *P < 0.05; **P < 0.01; ***P < 0.001; ****P < 0.0001. [file 12935_2023_2935_MOESM6_ESM.tif]

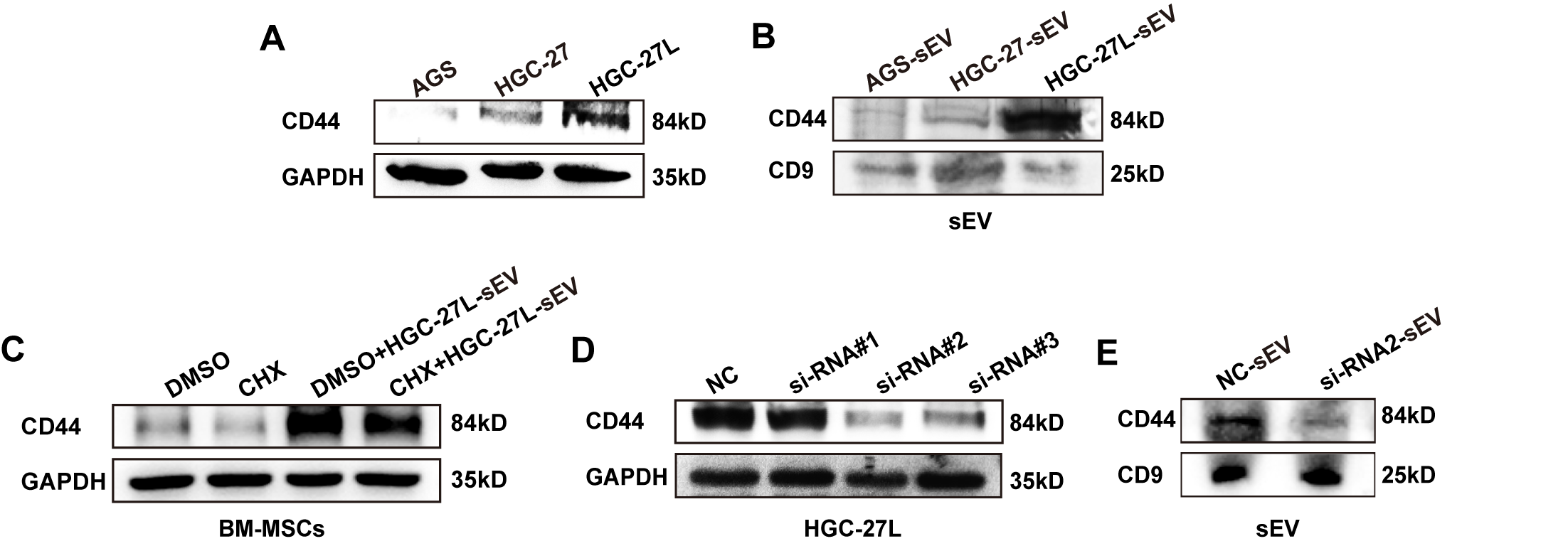

Supplement: Supplementary file 7 — Additional file 7. Fig. S3: CD44 is highly expressed in LNM-GCs and their sEV. A, B Comparison of cellular and sEV levels of the CD44 protein in GC cells by western blotting. C Effect of CHX on CD44 protein levels in BM-MSCs treated with or without HGC-27L-sEV. D Screening for the most efficient siRNA against CD44 in HGC-27L. E Western blotting analysis of CD44 contents in sEV derived from HGC-27L after transfection with siRNA and NC. [file 12935_2023_2935_MOESM7_ESM.tif]

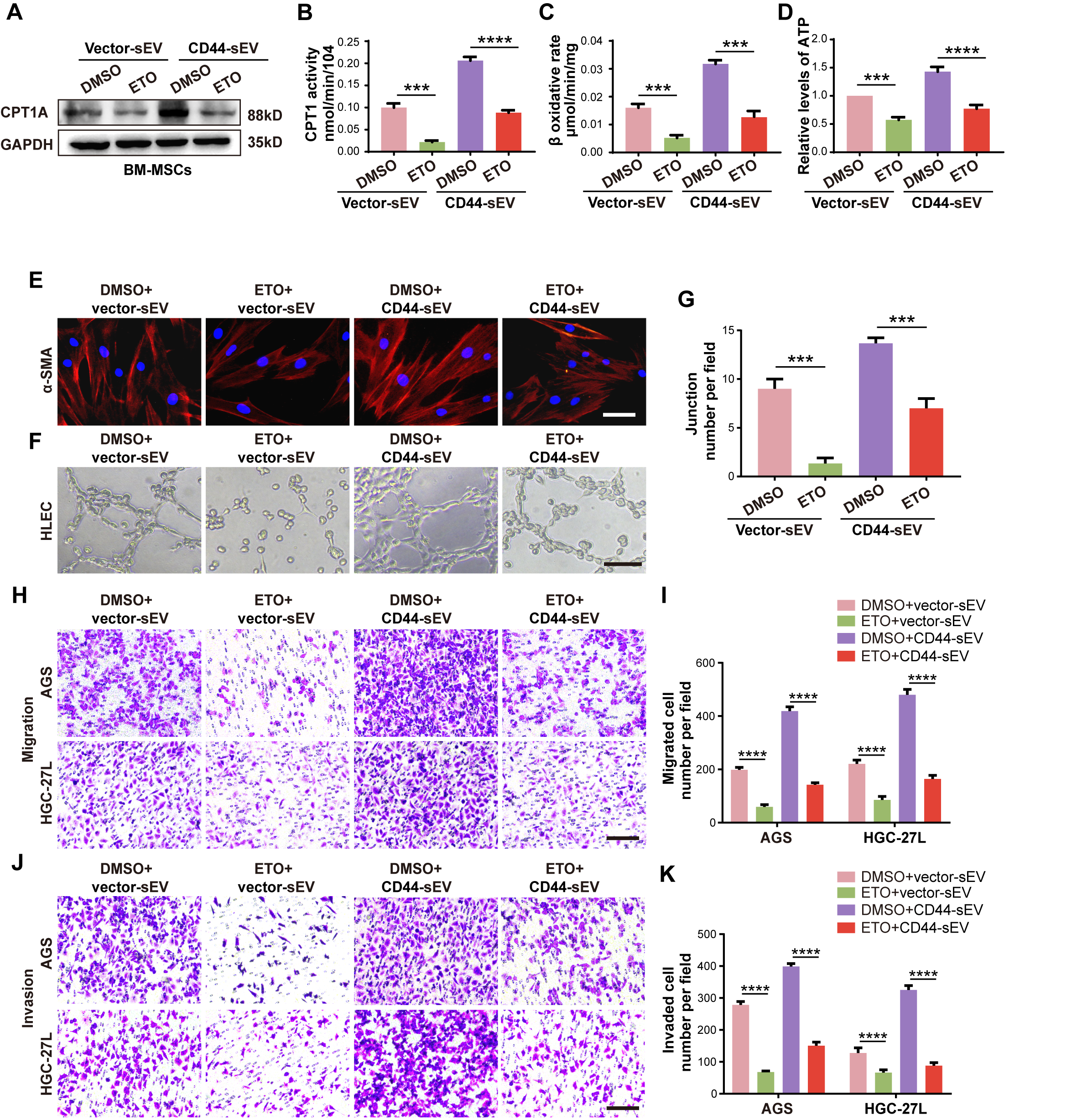

Supplement: Supplementary file 8 — Additional file 8. Fig. S4: Enhanced FAO is required for the education of BM-MSCs mediated by CD44. A Analysis of the effect of etomoxir on CPT1A expression in BM-MSCs treated with CD44-sEV using Western blotting. B–D The activity of CPT1, β-oxidation rate and ATP level detection in BM-MSCs. E Immunofluorescence staining for α-SMA. F, G Tubule formation assay. H–K Migration and invasion assays. ***P < 0.001; ****P < 0.0001. [file 12935_2023_2935_MOESM8_ESM.tif]

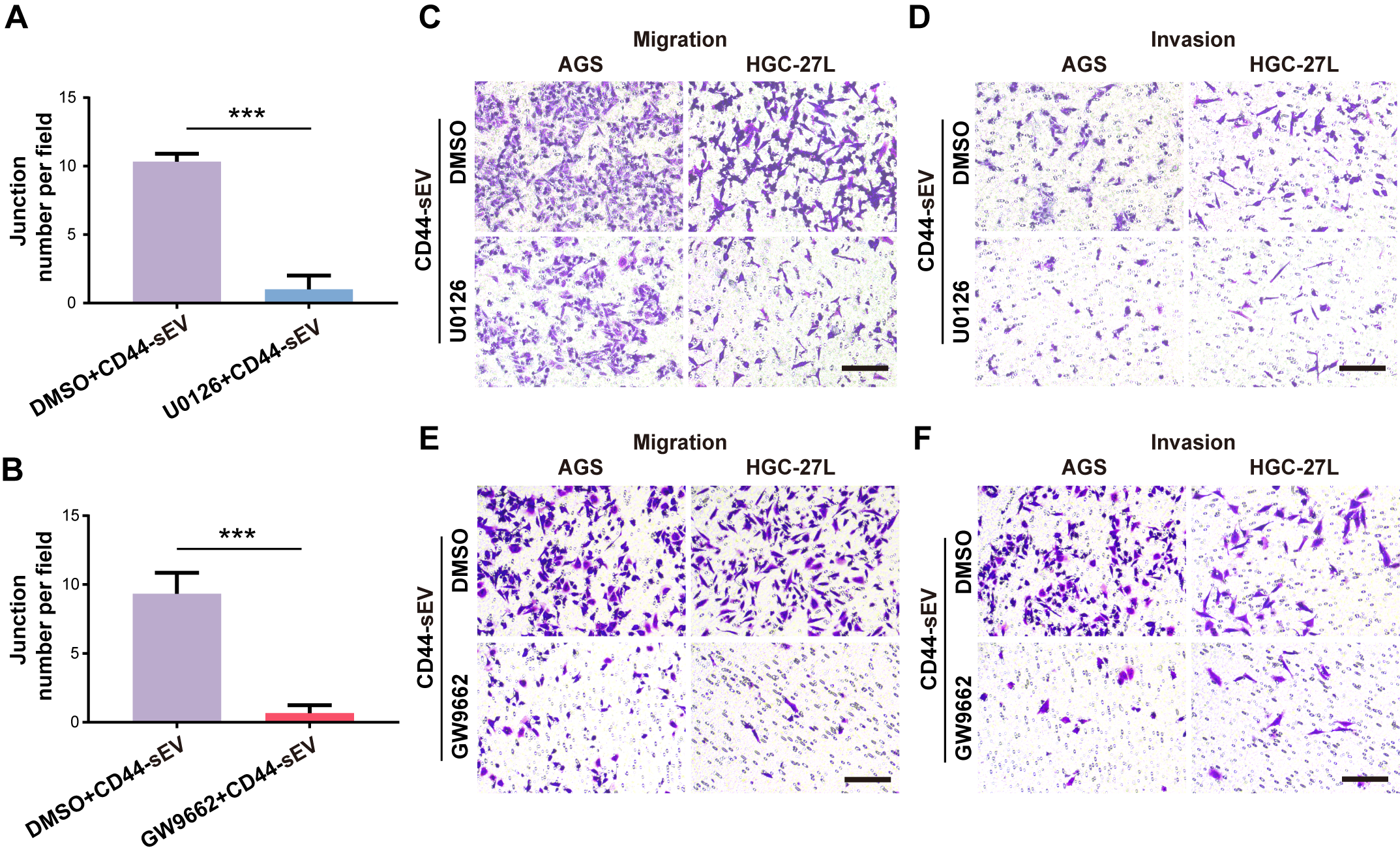

Supplement: Supplementary file 9 — Additional file 9. Fig. S5: U0126 and GW9662 suppress tumor-promoting capacity of BM-MSCs. A, B Quantification of formed tubule junctions. C–F Morphology of migrated and invaded cells. ***P < 0.001. [file 12935_2023_2935_MOESM9_ESM.tif]

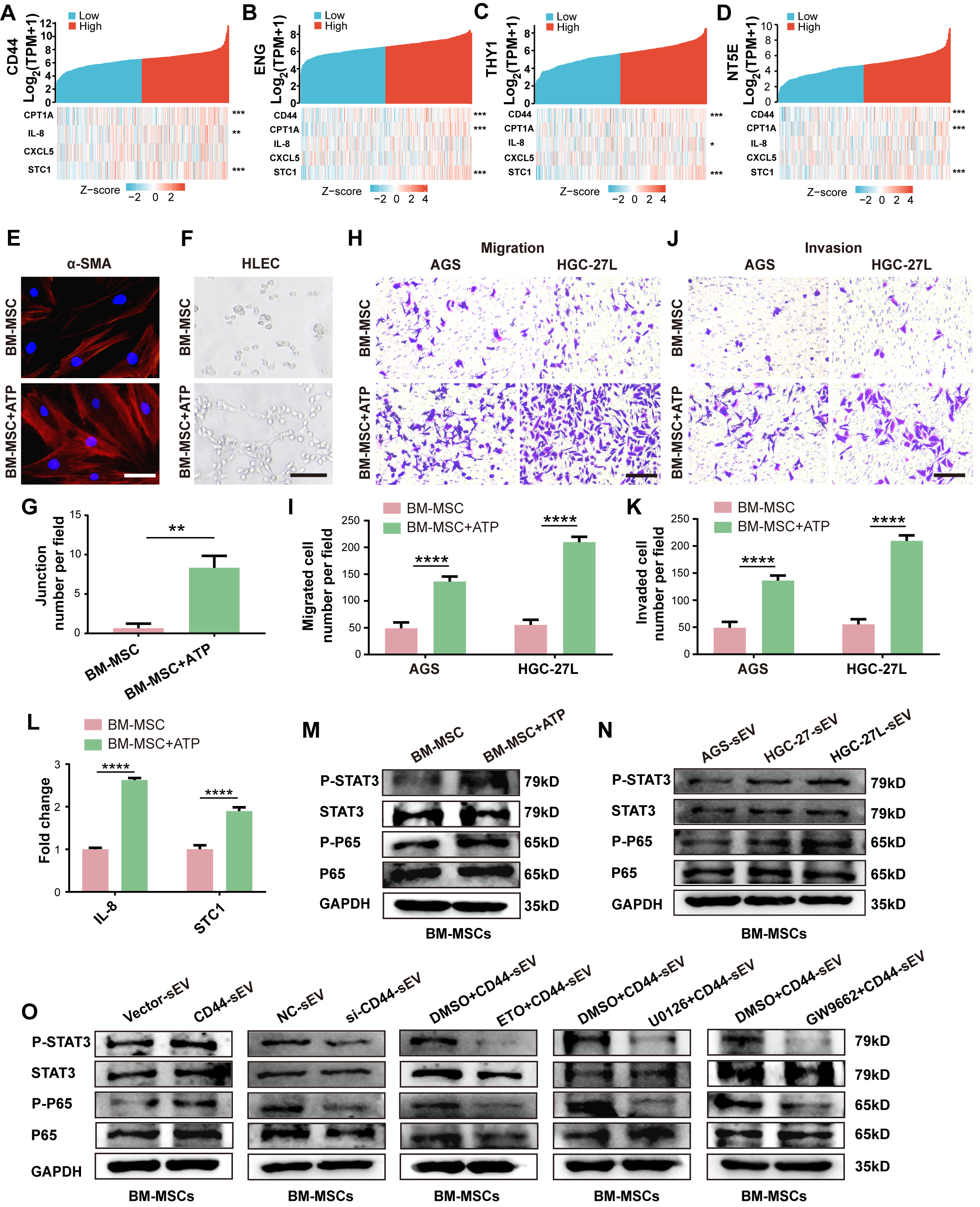

Supplement: Supplementary file 10 — Additional file 10. Fig. S6: ATP might be the effective metabolite of FAO to facilitate BM-MSCs education by activating STAT3 and NF-κB signaling. A Correlation of CD44 with CPT1A, IL-8, CXCL5 and STC1 were analyzed according to the data of TCGA-STAD. B–D Correlation of MSC markers ENG, THY1 and NT5E with CD44, CPT1A, IL-8, CXCL5 and STC1 in GC tissue from data of TCGA-STAD. E–K Effect of ATP on the education of BM-MSCs. E Immunofluorescence staining for α-SMA. F, G Tubule formation assay. H–K Migration and invasion assays. L The mRNA levels of IL-8 and STC1 in BM-MSCs with or without ATP were measured by RT-qPCR. M–O Western blotting analysis of p-STAT3 and p-P65 in BM-MSCs under indicated treatment. **P < 0.01; ****P < 0.0001. [file 12935_2023_2935_MOESM10_ESM.tif]

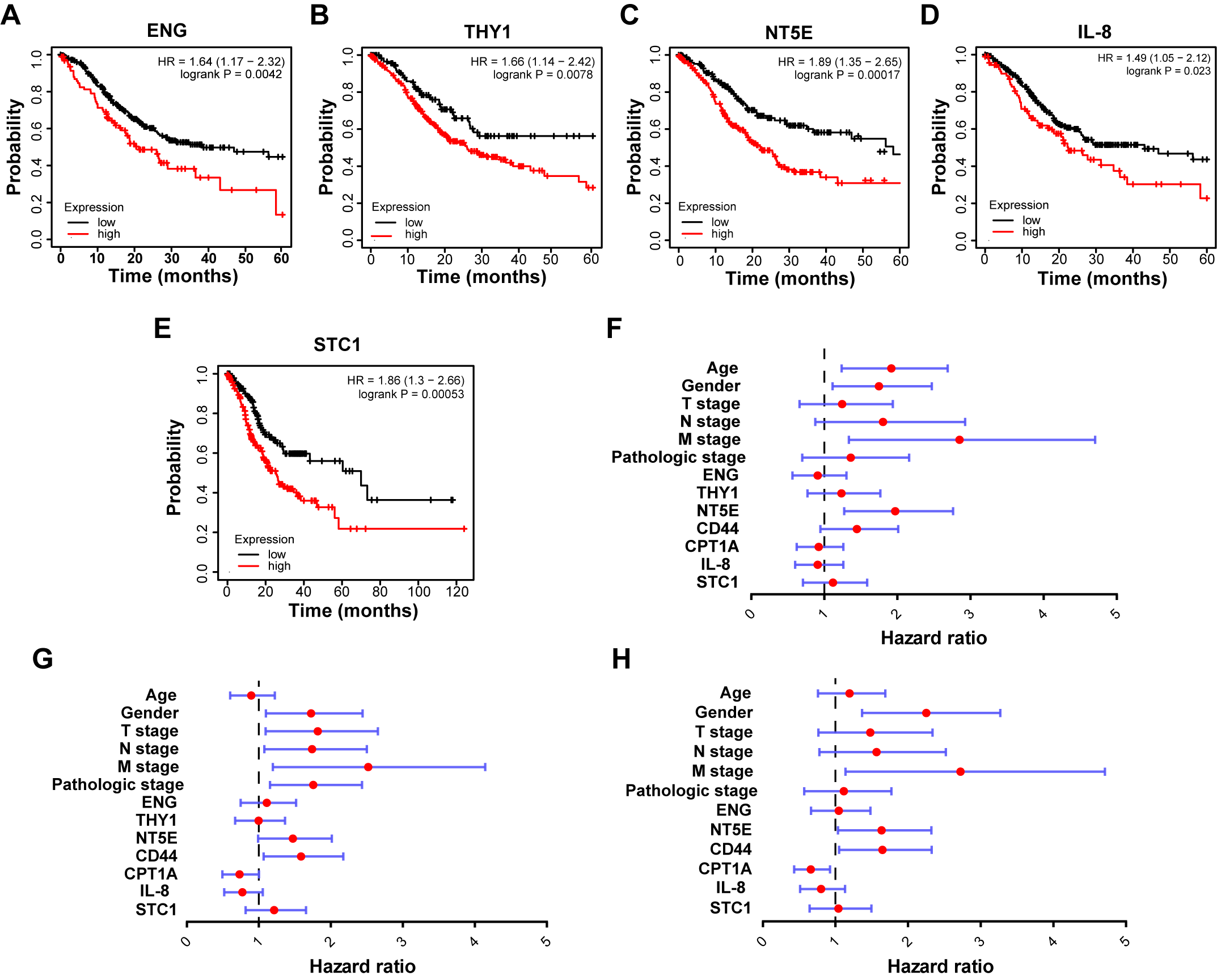

Supplement: Supplementary file 11 — Additional file 11. Fig. S7: High expression of MSC markers, CD44, CPT1A, CXCL8 and STC1 predict poor survival of GC. A–E Kaplan–Meier analysis of ENG, THY1, NT5E, IL-8 and STC1 association with OS of GC patients. F Forest plot of specific molecules effect on OS of GC patients. G, H Forest plot of specific molecules effect on FPS of GC patients. [file 12935_2023_2935_MOESM11_ESM.tif]
